# Comprehensive comparative analysis and development of molecular markers for *Lasianthus* species based on complete chloroplast genome sequences

**DOI:** 10.1186/s12870-024-05383-z

**Published:** 2024-12-31

**Authors:** Yue Zhang, Meifang Song, Deying Tang, Xianjing Li, Niaojiao Xu, Haitao Li, Lu Qu, Yunqiang Wang, Cuiyun Yin, Lixia Zhang, Zhonglian Zhang

**Affiliations:** 1 Yunnan Key Laboratory of Southern Medicine Utilization, Yunnan Branch of Institute of Medicinal Plant Development Chinese Academy of Medical Sciences, Peking Union Medical College, Jinghong, 666100 China; 2 College of Pharmacy, Dali University, Dali, 671000 China

**Keywords:** *Lasianthus*, Chloroplast genome, Species identification, Phylogenetic relationship, Rubiaceae

## Abstract

**Background:**

*Lasianthus* species are widely used in traditional Chinese folk medicine with high medicinal value. However, source materials and herbarium specimens are often misidentified due to morphological characteristics and commonly used DNA barcode fragments are not sufficient for accurately identifying *Lasianthus* species. To improve the molecular methods for distinguishing among *Lasianthus* species, we report the complete chloroplast (CP) genomes of *Lasianthus attenuatus*, *Lasianthus henryi*, *Lasianthus hookeri*, *Lasianthus sikkimensis*, obtained via high-throughput Illumina sequencing.

**Results:**

These showed CP genomes size of 160164-160246 bp and a typical quadripartite structure, including a large single-copy region (86675–86848 bp), a small single-copy region (17177–17326 bp), and a pair of inverted repeats (28089–28135 bp). As a whole, the gene order, GC content and IR/SC boundary structure were remarkably similar among of the four *Lasianthus* CP genomes, the partial gene length and IR, LSC and SSC regions length are still different. The average GC content of the CP genomes was 36.71–36.75%, and a total of 129 genes were detected, including 83 different protein-coding genes, 8 different rRNA genes and 38 different tRNA genes. Furthermore, we compared our 4 complete CP genomes data with publicly available CP genome data from six other *Lasianthus* species, and we initially screened eleven highly variable region fragments were initially screened. We then evaluated the identification efficiency of eleven highly variable region fragments and 5 regular barcode fragments. Ultimately, we found that the optimal combination fragment' ITS2 + *psa*I-*ycf*4' could authenticated the *Lasianthus* species well. Additionally, the results of genome comparison of Rubiaceae species showed that the coding region is more conservative than the non-coding region, and the *ycf*1 gene shows the most significant variation. Finally, 49 species of CP genome sequences belonging to 16 genera of the Rubiaceae family were used to construct phylogenetic trees.

**Conclusions:**

Our research is the first to analyze the chloroplast genomes of four species of *Lasianthus* in detail and we ultimately determined that the combination fragment' ITS2 + *psa*I-*ycf*4' is the optimal barcode combination for identifying the genus of *Lasianthus*. Meanwhile, we gathered the available CP genome sequences from the Rubiaceae and used them to construct the most comprehensive phylogenetic tree for the Rubiaceae family. These investigations provide an important reference point for further studies in the species identification, genetic diversity, and phylogenetic analyses of Rubiaceae species.

**Supplementary Information:**

The online version contains supplementary material available at 10.1186/s12870-024-05383-z.

## Introduction

*Lasianthus* is a large genus with more than 200 species in family Rubiaceae [1]. These plants have the effect of promoting blood circulation and alleviating pain, and they are used in several traditional Chinese folk medicines to treat conditions such as fever, blood loss and bone pain with *L. lucidus* [2], and *L. hookeri* can be used as a food for promoting blood circulation [3]. The root decoction of *L. oblongus* is applied orally to hasten constriction of the organs for postpartum mothers [4]. *L. acuminatissimus* is used in traditional Chinese folk medicine for the treatment of rheumatoid arthritis [5]. In addition, researchers used various chromatographic methods to isolate and identify secondary metabolites of the *Lasianthus* species. These included antitumor anthraquinone glycosides isolated from *L. acuminatidis*, five new iridoid glycosides isolated from *L. verticillatus*, and iridoid terpenoids isolated from *L. attenuatus* [5–7]. However, source materials and herbarium specimens are often not well explored due to the similarities in morphological characters among *Lasianthus* species and their medicinal parts. This has further led to a chaotic situation in the Chinese folk medicinal market, characterized by the cross mixing of different original medicinal materials. These problems have severely hindered the clinical use of scientific research related to medicinal *Lasianthus* species.

Most of the *Lasianthus* species are shrubs, including a few small trees [8]. At present, the classification of *Lasianthus* species is mainly focused on microscopic and macroscopic morphological identification [8, 9]. However, with the change in growth environment, the microscopic morphology of plants also changes slightly, so morphological classification is difficult to identify the species of *Lasianthus* accurately. Studies on molecular identification of *Lasianthus* species are scarce, only Arshed et al. [10] reported evaluating the feasibility of five candidate DNA barcoding loci for Philippine *Lasianthus* Jack., and the results indicate that ITS, *mat*K, *rbc*L, *rps*16 and *trn*T-F markers could not accurately identify all *Lasianthus* species. These results indicate that commonly used DNA barcoding sequences are not sufficient for accurately identifying the *Lasianthus* species.

Chloroplasts (CP) are important organelles of photosynthesis in green plants. The chloroplast gene is a closed circular DNA molecule composed of a typical quadripartite structure: a large single-copy region (LSC), a small single-copy region (SSC) and a pair of mirrored inverted repeat sequences (IRa and IRb) [11–13]. Chloroplast genomes are often used for species identification, systematics research, and the development of molecular markers because of their stable structure, maternal clonal inheritance, and low genetic recombination rate [14, 15]. Many chloroplast gene fragments such as *trn*H-*psb*A, *mat*K and *rbc*L are used as DNA barcodes for species identification. However, it is difficult to identify related species by common fragments alone [16]. With the rapid development of high-throughput sequencing technology in recent years, the complete CP genome sequence becomes easy to obtain. The whole CP genome as a super-barcode has been widely used in plant phylogenetic relationship evaluation or species identification, and the sequences selected from the highly-variation regions of the whole CP genome have been used for species identification [17, 18]. For example, Yang et al. [18] conducted plant phylogenetic analysis and molecular marker development based on chloroplast whole genome sequencing of five medicinal plants in the genus *Alpinia*. Zhang et al. [19] developed barcode markers by comparing the complete CP genome sequences of *Dracaena* species to aid in the accurate identification of the origin of Dragon’s blood (*Dracaena*) medicinal herbs. However, there is limited publicly available data for *Lasianthus*. Although *L. attenuatus*, *L. hookeri*, *L. sikkimensis*, *L. chrysoneurus*, *L. japonicus*, *L. rigidus*, *L. verticillatus* have published the fasta format sequences of the chloroplast genome in NCBI, but these sequences have not been properly annotated. Relative to other families and genera in the plant kingdom, the CP genome data of *Lasianthus* plants are very limited. Therefore, it is necessary to obtain more CP genome data to solve the small intraspecific and interspecific differences among species of *Lasianthus*, to support the effective utilization of medicinal plant resources.

Here, we sequenced the complete CP genomes of *L. attenuatus* sampled from Guangxi and *L. henryi*, *L. hookeri*, *L. sikkimensis* sampled from Yunnan, using the Illumina HiSeq4000 sequencing platform. We also investigated their basic characteristics: including molecular structure analysis, simple sequence repeats (SSRs) and long repeat sequence analysis. Next, we compared the chloroplast genomes of *Lasianthus* species, analyzed nucleotide diversity, and identified hypervariable regions to develop DNA markers. Then, we collected 35 samples from 7 *Lasianthus* species to verify the identification efficiency of molecular markers and found the optimal identification fragments. Finally, we collected 49 species CP genome sequences from the Rubiaceae family and used them as a super-barcode to identify the species in this group and analyzed their phylogenetic relationships. This study provides important genetic information for species identification and phylogenetic analysis of *Lasianthus*. At the same time, it is also helpful to alleviate the problem of accurate identification of *Lasianthus* plants in the medicinal material market.

## Materials and methods

### Sample collection and DNA extraction

Fresh young leaves were collected from the *L. attenuatus* growing in Guangxi and *L. henryi*, *L. hookeri*, *L. sikkimensis* growing in Yunnan. The voucher specimens were deposited in the herbarium, Yunnan branch of the Institute of Medicinal Plant Development (IMPLAD), Chinese Academy of Medical Sciences herbarium (voucher numbers: IMDY2022051002, IMDY2022091311, IMDY2021102605, IMDY2021110615) and identified by Zhonglian Zhang. The collected leaves were cleaned with 75% ethanol, transported in dry ice, and preserved at -80 ° C for plant DNA extraction. Using the TIANGEN plant Genomic DNA kit (Tianjin Biotech, Beijing, Co., Ltd.) to extract total genomic DNA from frozen leaves according to a standard protocol. The concentration and quality of total DNA were evaluated using electrophoresis in 1% (w/v) agarose gel and Nanodrop 2000 instrument (Thermo Fisher Scientific Inc., Waltham, MA, USA). The OD260/280 value ranges from 1.8 to 2.2, and ≥ 2 µg of was equally pooled from individuals of the four species could be used to construct the library.

### Chloroplast genome sequencing, assembly and annotation

DNA was broken into 300–500 bp fragments using the Covaris M220 focused ultrasonicator (Covaris, Woburn, MA, United States), and fragments of 500 bp size were screened for library construction. The DNA library was constructed using the Illumina TruSeq™ Nano DNA Sample Prep Kit (Illumina, San Diego, CA, United States). The library enrichment was performed by eight cycles of polymerase chain reaction (PCR) amplification, and the target band was recovered from 2% agarose gel (Certified Low Range Ultra agarose). The library was sequenced using the Illumina HiSeq4000 sequencing platform at Biozeron Company (Shanghai, China), and 2 × 150 bp paired-end reads were obtained. Raw reads were checked(Q ≥ 25) using the FastQC Toolkit [20]. Low-quality reads were filtered out from the raw data, reads containing 10% N were removed, and small fragments of < 75 bp were discarded after high-quality pruning to obtain high-quality data (clean reads) for subsequent analysis. Then, the above data was uploaded to the server with FileZilla 3.51.0, and the chloroplast genome was De novo assembled using Get Organelle [21]. The filtered ‘gfa’ file was visualized in Bandage v.0.8.1 [22]. Next, Bowite 2 in Geneious v.8.0.2 [23] was used to align the raw sequence to the assembled chloroplast genome to verify the assembly results. Finally, the reference genome was used to correct the starting position of the CP assembly sequence, and determine the position and direction of the four CP regions (LSC, IRa, SSC, and IRb) to obtain the assembled CP genome sequence.

The assembly results were imported into Geneious v.8.0.2 [23] for annotation, and then the positions of start codon, stop codon and intron of protein-coding genes were manually adjusted in Geneious v.8.0.2 [23]. The tRNA gene was validated online using the tRNAscan SE service [24]. The chloroplast genome map was drawn using the online website (https://chlorobox.mpimp-golm.mpg.de/OGDraw.html)) [25]. Finally, we obtained the sqn file and submitted our report to NCBI. The complete CP genome sequences of *L. attenuatus*, L. *henryi*, *L. hookeri* and *L. sikkimensis* were deposited in GenBank with accession numbers of OR490208, OR490209, OR490210 and OR490211, respectively.

### Codon usage and repeat sequence analysis

CodonW software (University of Texas, Houston, TX, usa) was used to obtain relative synonymous codon usage (RSCU) and investigate the codon distribution [26]. Molecular Evolutionary Genetics Analysis Version X was used to analyze guanine-cytosine (GC) content [27]. Simple sequence repeats were detected using the MISA Perl Script (http://pgrc.ipk-gatersleben.de/misa/). The minimum number of repeat units was set as follows: 10 repeat units for mononucleotide repeats, 5 for di-nucleotide repeats, 4 for tri-nucleotide repeats, and 3 for tetra-, penta-, and hexanucleotide repeats. REPuter was used to detect *L. attenuatus*, *L. henryi*, *L. hookeri* and *L. sikkimensis* of long repeats, including forward, palindromic, reverse, and complementary repeats [28].

### Genome comparison analyses and marker development

The whole CP genomes were initially aligned using the online MAFFT software [29]. Conserved sequences between the CP genomes of *L. attenuatus*, *L. henryi*, *L. hookeri* and *L. sikkimensis* were identified using BLASTN with an E-value cutoff of 1e-10. The mVISTA [30] program in Shuffle-LAGAN mode was used to compare the four *Lasianthus* CP genomes using the *L. henryi* CP genome as a reference. Then, we used DnaSP [31] software to determine the nucleotide diversity (Pi) with a 200 bp step size and a 600 bp window length.

We used the primer design tool Primer-BLAST to design labeled primers for the highly variable regions (http://www.ncbi.nlm.nih.gov/tools/primer-blast/). This enabled verification of interspecies polymorphisms in the CP genomes and the development of DNA markers to identify *Lasianthus* species via genomic comparisons and analyses. Next, we obtained seven species of *Lasianthus* (*L. attenuatus*, *L. henryi*, *L. hookeri*, *L. sikkimensis*, *L. fordii* var. *trichocladus*, *L. hookeri* var. *dunnianus* and *L. verticillatus*) to validate the efficiency of DNA barcoding based on the selected highly variable regions. The sample number and location information are listed in Table S1. Total genomic DNA was extracted using the TaKaRa MiniBEST Universal Genomic DNA Extraction Kit with a standard protocol (TaKaRa) and 1% agarose gel electrophoresis. We used an ultra-micro ultraviolet spectrophotometer to assess the purity and concentration of the extracted genomic DNA. The PCR reactions were conducted in a total reaction volume of 25 µL, which contained DNA (15 ng), 10× PCR buffer (2.5 µL), dNTPs (10 mM, 2 µL), primers (0.5 µL each), Taq DNA polymerase (5 U/µL, 0.5 µL; TaKaRa), and double-distilled water (18.5 µL). For each reaction, we used the following program: an initial 5 min of denaturation at 94℃; 35 cycles of 30 s at 94℃, 30 s of annealing at Tm with different primers, and 15 s of extension at 72℃; and a final extension for 7 min at 72℃. The PCR products were visualized using 1.5% agarose gels, and the successfully amplified PCR products were sent to Sangon Biotech (Shanghai, China) for bidirectional sequencing.

### Phylogenetic analysis

To determine the phylogenetic positions of *L. attenuatus*, *L. henryi*, *L. hookeri* and *L. sikkimensis*, we downloaded 63 complete CP genomes of Rubiaceae from the NCBI database. The sequences were initially compared using MAFFT [29]. We also used the CP genomes of *Lonicera gynochlamydea* (NC_064373), *L. similis* (NC_060471), and *Sambucus williamsii* (MW788534) as outgroups. We constructed phylogenetic trees of CP genomes sequences of Rubiaceae family species using the Neighbor-Joining (NJ), Maximum Parsimony (MP) and Maximum Likelihood (ML) methods with MEGA X [27] software and 1000 bootstrap replicates, and the best-fit substitution models were selected by ModelTest-NG [32].

## Results and discussion

### Chloroplast genome features of *Lasianthus* species

**Fig. 1:**
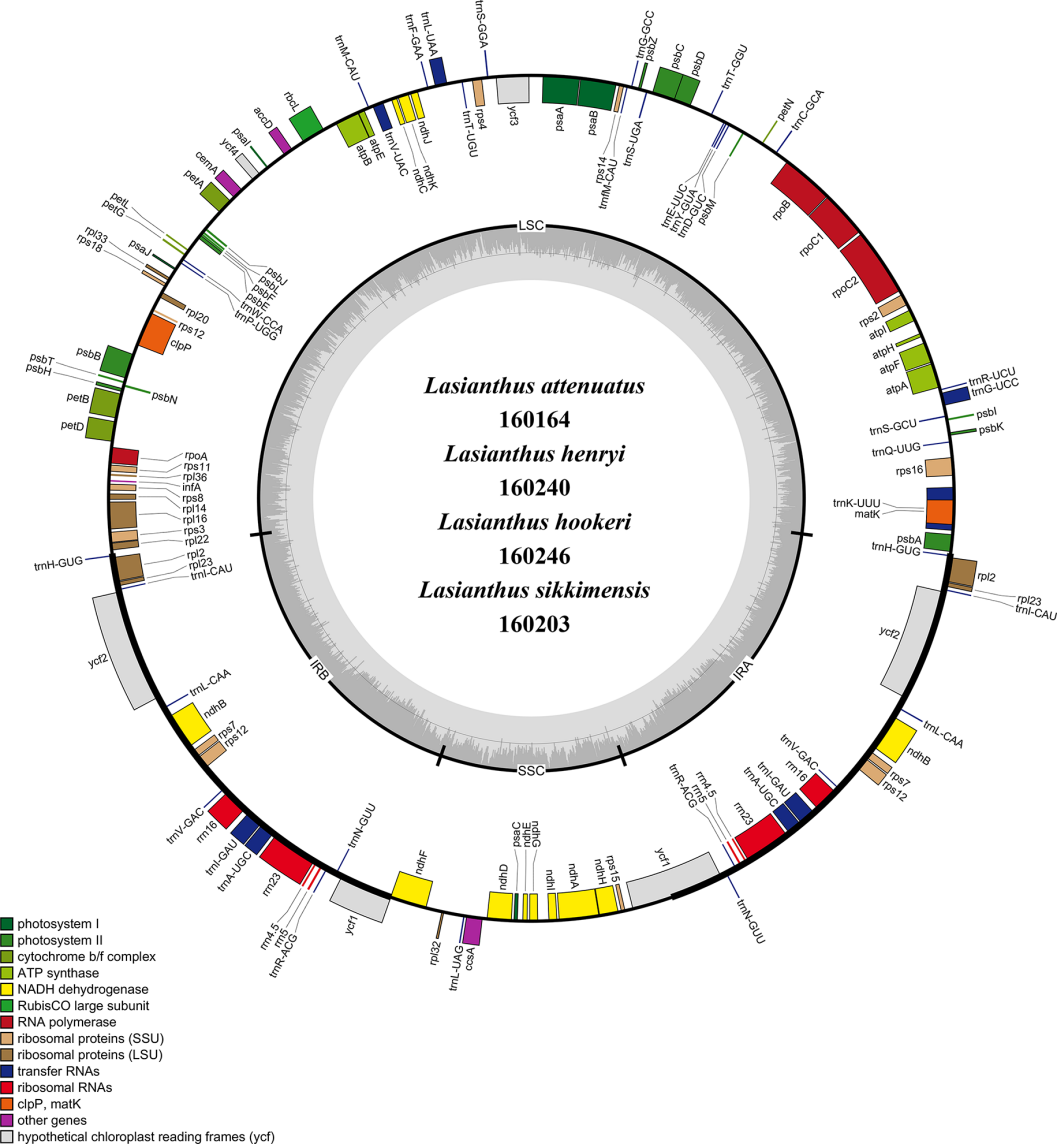
Gene map of four *Lasianthus* complete chloroplast genomes. Genes on the inside of the outer circle are transcribed in a clockwise direction, while genes on the outside of the outer circle are transcribed in a counterclockwise direction. Genes belonging to different functional categories are different color-coded. The inner circle indicates the range of the LSC, SSC, and IRs. Also, the darker gray area in the inner circle corresponds to the GC content, whereas the lighter gray area corresponds to the AT content

We analyzed and compared the basic characteristics of four *Lasianthus* species. The results showed that the CP genomes of *L. attenuatus*, *L. henryi*, *L. hookeri* and *L. sikkimensis* have the typical quadripartite structures [13, 16, 18] with a genome size of 160,164 bp, 160,240 bp, 160,246 bp and 160,203 bp, respectively (Fig. 1). Among them, the chloroplast genome of *L. hookeri* is the longest and *L. attenuatus* is the shortest, with a difference of only 82 bp. The chloroplast genome of *Lasianthus* species has a typical four-region structure like other higher plants [13, 33]. It contained a LSC region (86,675–86,848 bp), an SSC region (17,177–17,326 bp) and a pair of IRs (28,089–28,135 bp). The CP genomes of four *Lasianthus* species encodes a total of 129 genes, including 83 different protein-coding genes, 8 different rRNA genes and 38 different tRNA genes. Similar results have been reported in other angiosperms. The GC content of CP genome of the four *Lasianthus* species is very similar, ranging from 36.71 to 36.75% (Table 1).

**Table 1:** Summary of chloroplast genome characteristics of four *Lasianthus* chloroplast genomes

Species names	*L. attenuatus*	*L. henryi*	*L. hookeri*	*L. sikkimensis*
GenBank accession	OR490208	OR490209	OR490210	OR490211
Size (bp)	160,164	160,240	160,246	160,203
LSC length (bp)	86,751	86,675	86,742	86,848
SSC length (bp)	17,229	17,295	17,326	17,177
IR length (bp)	28,092	28,135	28,089	28,089
Coding (bp)	77,094	77,091	77,100	76,941
Non-coding (bp)	83,070	83,149	83,146	83,262
GC content (%)	36.72	36.74	36.71	36.75
Total genes	129	129	129	129
Protein-coding genes	83	83	83	83
tRNA genes	38	38	38	38
rRNA genes	8	8	8	8

### Codon usage

**Fig. 2:**
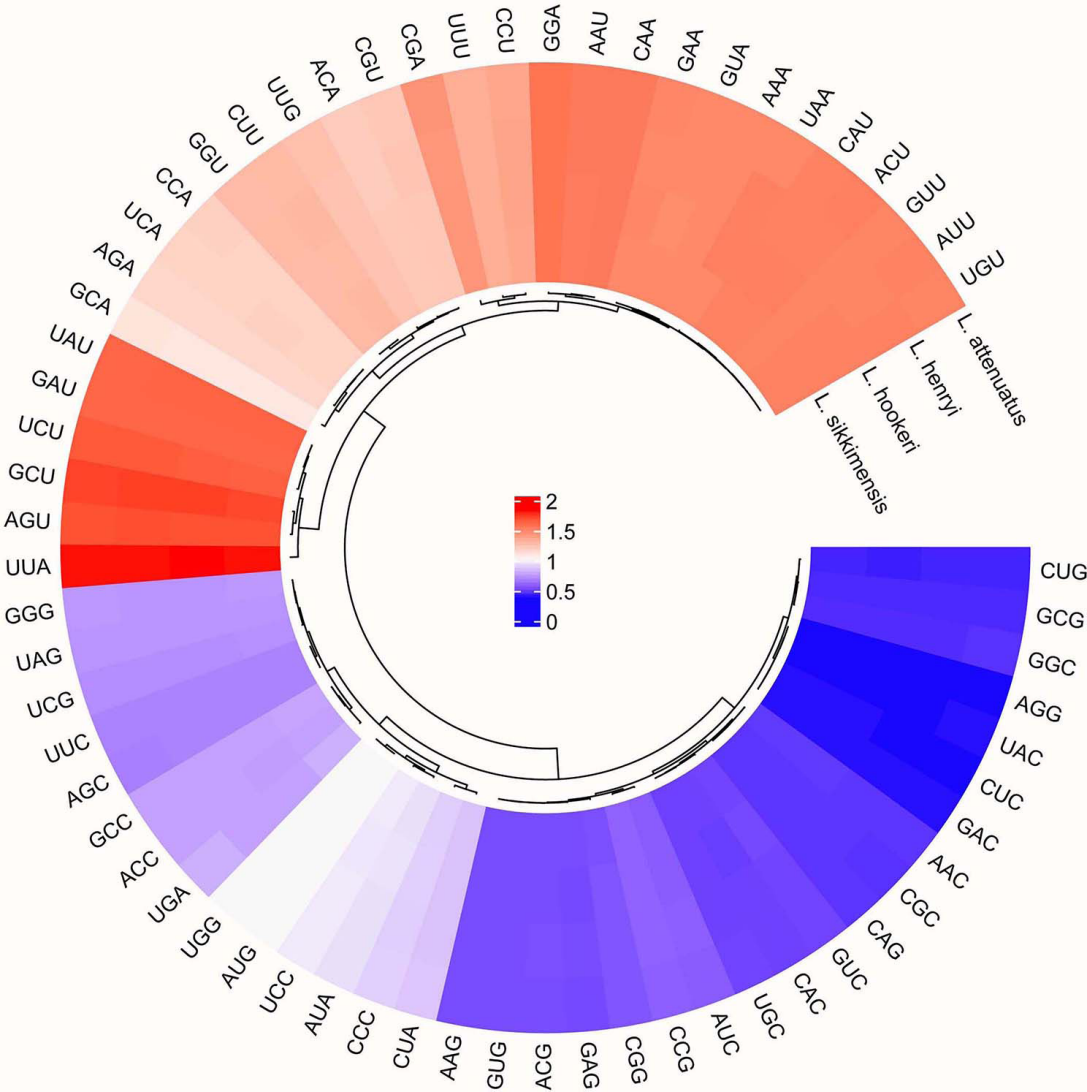
Heatmap of codon distribution of common protein-coding genes of four *Lasianthus* species

Codon usage bias plays an important role in CP genome evolution [34]. Some researchers pointed out that natural selection, mutation, phylogenetic relationship and other factors may lead to different codon use preferences [34–36]. The relative synonymous codon usage (RSCU) ratio is used to measure the usage of synonymous and non-synonymous codons in coding sequences. When the RSCU ratio < 1.00, the frequency of codon usage is lower than expected, and when the RSCU ratio > 1.00, the frequency of codon usage is higher than expected [26, 37]. We analyzed the codon usage levels of the shared protein-coding genes in the four *Lasianthus* species CP genomes (Fig. 2, Table S2). In total, the genes in the *L. attenuatus*, *L. henryi*, *L. hookeri* and *L. sikkimensis* CP genomes contain 25,698, 25,697, 25,700 and 25,647 codons, respectively. The codon for leucine is the most common in the four *Lasianthus* species CP genomes, accounting for 10.8% of the total number of codons on average. In the CP genomes of these *Lasianthus* species, usage of the codons AUG and UGG (encoding methionine and tryptophan, respectively) is not biased (RSCU ratio = 1.00). The AUG is also the initiator codon used by most protein-coding genes in the CP genome of terrestrial plants. Most amino acids were coded by more than one synonymous codon, such as leucine and arginine, which encode six codons. Only methionine and tryptophan do not have alternative codons. In the CP genome of higher terrestrial plants, the preference of the third base of the codon for A / T(U) is generally higher than C / G [38, 39]. In this study, codons ending in A and/or U accounted for 69.29–69.37% of all protein-coding genes in the CP genomes of four *Lasianthus* species. Moreover, these codons typically have high RSCU ratios in the four CP genomes, such as UUA (1.81–1.82) encoding leucine, GCU (1.71–1.73) encoding alanine. These codon usage results are similar to with those previously reported for *Saxifraga* species, *Cardamine hupingshanensis*, *Alpinia galanga* and *Alpinia kwangsiensis* [34, 37, 40]. Our results also showed that all types of RSCU ratio > 1.00 in the four *Lasianthus* species end with A or U except Ile-AUA and Leu-CUA. The high RSCU ratio may be related to the function of amino acids or the structure of peptides required to avoid transcription errors during the evolution of the CP genome [41, 42]. Therefore, stable CP genome evolution helps to reduce harmful mutations while improving the adaptability of important CP genes to selection pressure [37, 43, 44].

### Analyses of simple sequence repeats and long repeats

Simple sequence repeats (SSRs) are tandem repeat sequences composed of 1–6 nucleotide repeat units, widely distributed in the protein-coding genes [15, 45]. We analyzed the distribution and types of SSRs in CP genomes of four *Lasianthus* species. A total of 68,63,60,63 SSRs were found in the *L. attenuatus*, *L. henryi*, *L. hookeri* and *L. sikkimensis* CP genomes using MISA software, respectively (Table 2). Among these repeats, the mononucleotide SSRs were the most abundant, which were found (25–32) times in the four *Lasianthus* species. Followed by trinucleotide (9–15), tetranucleotide (8–9), pentanucleotide (7–8) and dinucleotide (6–7) repeats. Furthermore, the main repeats were constituted by A/T (21–27) in the four *Lasianthus* species, followed by AAT/ATT (8–14) trinucleotide repeats, AT/AT (5–6) dinucleotide repeats and AAAT/ATTT (5–6) tetranucleotide repeats. Our results are consistent with previous studies reporting that the Chloroplast genome SSRs are mostly composed of polyadenine ( Poly-A ) or polythymine ( Poly-T ) repeat, and the contents of C and G repeat are rare, which is consistent with the general characteristics of chloroplast genome SSRs in many plants [46–48].Due to the high substitution rate of CP SSRs, SSRs markers are widely used in genetic diversity and population structure evaluation, marker-assisted selection breeding, genetic map development and germplasm resources of plant populations [37, 49, 50].

**Table 2:** The simple sequence repeats (SSRs) types of the four CP genomes of *Lasianthus* species

SSR type	Repeat unit	Amount			
		*L. attenuatus*	*L. henryi*	*L. hookeri*	*L. sikkimensis*
Mono	A/T	23	27	22	21
	C/G	7	5	4	4
Di	AC/GT	1	1	1	1
	AT/AT	5	5	6	6
Tri	AAT/ATT	14	8	8	11
	AGC/CTG	1	1	1	1
Tetra	AAAG/CTTT	1	1	1	1
	AAAT/ATTT	5	5	6	6
	AATT/AATT	2	2	2	2
penta	AAAAC/GTTTT	1	0	1	1
	AAAAG/CTTTT	1	1	1	1
	AAAAT/ATTTT	5	5	5	4
	AATAT/ATATT	1	0	1	2
	AAATT/AATTT	0	1	0	0
Hexa	AAGATT/AATCTT	1	0	0	1
	AAGAAT/ATTCTT	0	1	1	1

**Fig. 3:**
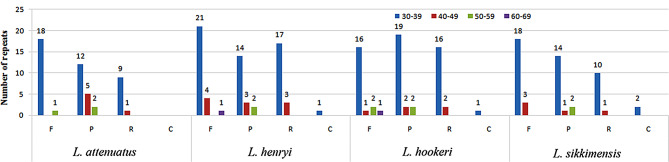
Long repeat sequence analysis of four *Lasianthus* complete chloroplast (CP) genomes. F, P, R, and C indicate the forward, palindromic, reverse, and complementary repeat types, respectively. Repeats with different lengths are indicated by different colors

Long Repeats sequences include four types: complementary, forward, reverse, and palindromic repeat [28, 37]. These repetitive structures help facilitate the molecular recombination and diversity analysis of the CP genome in the population [51]. In this paper, we detected complementary, forward, reverse, and palindromic repeats in four *Lasianthus* species CP genomes using REPuter software tools. Results of the Long repeat-sequence analysis is shown in Fig. 3. The results showed that *L. attenuatus* has the least number of repeats, including 19 forward, 19 palindromic and 10 reverse repeats. The number of forward repeats (19–26) was the most abundant, followed by palindromes (17–23) and reverse repeats (10–20), with complementary repeats (0–2) being the least abundant. *L. attenuatus* does not have any complementary repeat types. Among the four *Lasianthus* species, the length of these repeat sequences is mostly between 30 and 39 and 40–49 bp, with none exceeding 70 bp. Repeat sequences play an important role in genome rearrangement and recombination [52, 53]. The repeat sequences identified in this study provide useful resources for species identification, genetic diversity and population structure of *Lasianthus*.

### Contraction and expansion of IRs

**Fig. 4:**
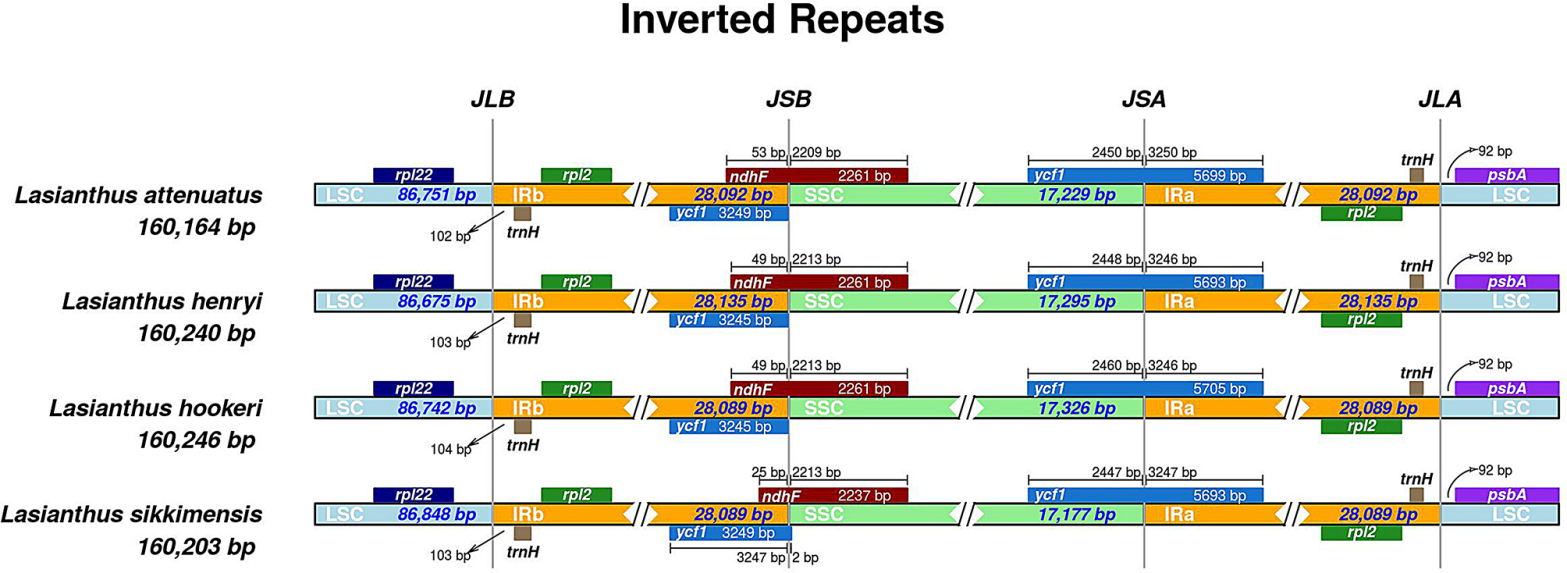
Comparison of the borders of the LSC, SSC, IRs regions among four CP genomes of *Lasianthus*. JLB: boundary of the LSC and the IRb. JSB: boundary of the SSC and the IRb. JSA: boundary of the SSC and the IRa. JLA: boundary of the LSC and the IRa

The chloroplast genome of angiosperms is highly conserved. Some researchers believe that the contraction and expansion of the boundary between the IR and LSC/SSC regions are the main reasons for the size change of the chloroplast genome [48, 54]. In this study, we compared the IR / LSC and IR / SSC boundary structures of four *Lasianthus* species. The expansion and contraction of the IR regions are shown in Fig. 4. The results showed that there was no significant difference among the four *Lasianthus* species in terms of the length range of IR regions, which was 28,089–28,135 bp. The *psb*A gene of all *Lasianthus* species was identical in location, as it was completely located in the LSC region and was 92 bp away from the IRa / LSC boundary. The *ndh*F encoding gene located at the IRB-SSC boundary, and the ndhF gene has a length of 25–53 bp in the IRb region. Furthermore, all the IRa regions expand 3246–3250 bp into *ycf*1 and form a pseudogene *ycf*1 with a length of 3245–3249 bp in the IRb region. This also resulted in a 25–53 bp overlap between the pseudogene *ycf*1 and the gene *ndh*F in the IRb region. The pseudogenization of *ycf*1 and the location of ycf1 copies were also frequently found in other plants [55, 56]. In summary, although the chloroplast genomes of the four species are well conserved including gene number and genomic structures, the partial gene length and IR, LSC and SSC regions length are still different. This phenomenon indicates suggested expansions and contractions of the IR regions, as contraction and expansion of the IR/ SC boundary are considered to be the main reason for the length change of the chloroplast genome [38, 54, 57]. This is also a driving force in plant CP genome variation [58].

### Comparative genomic analysis within *Lasianthus*

**Fig. 5:**
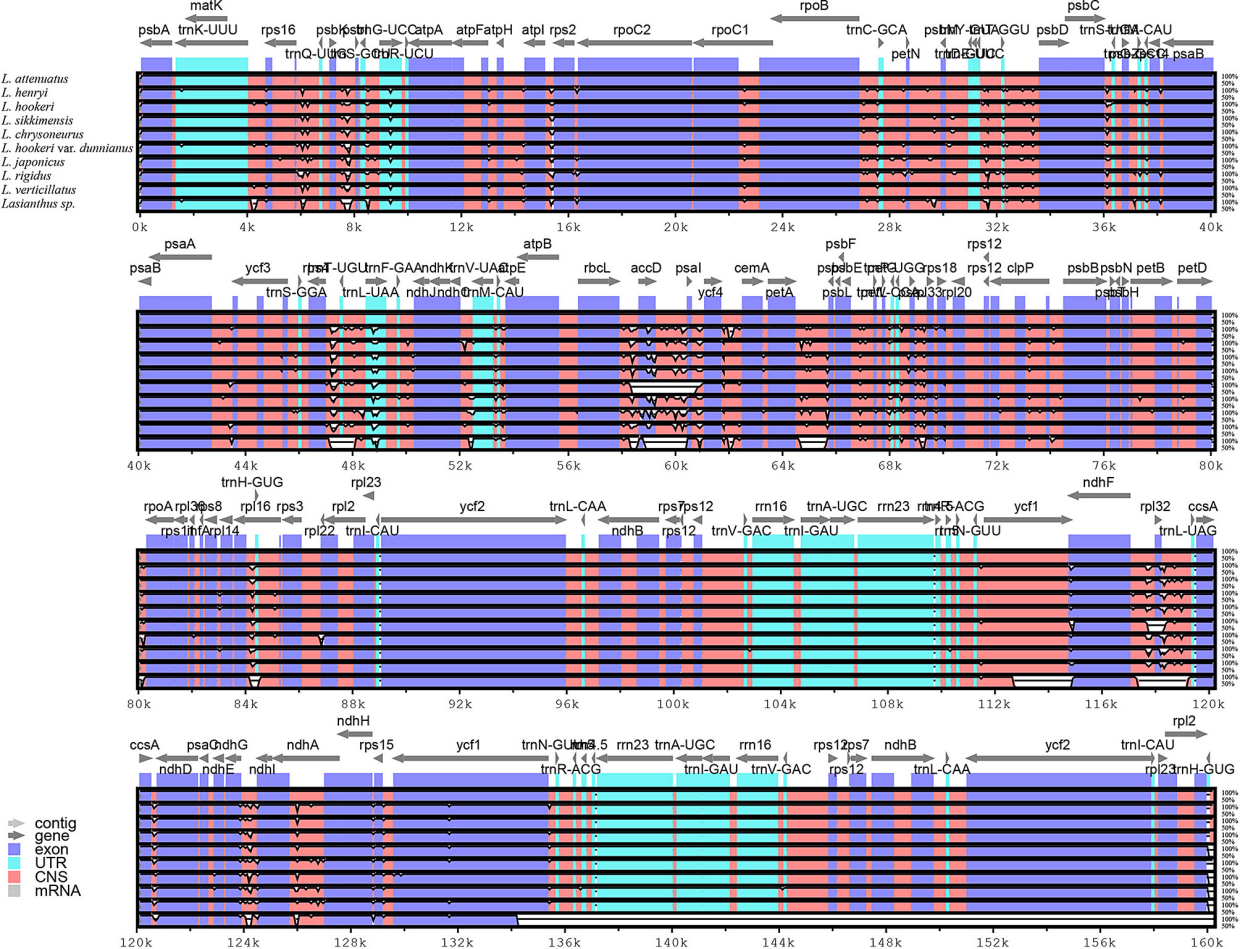
The alignment and comparative analysis of the whole CP genome for ten *Lasianthus* species using mVISTA, and using *L. attenuatus* as a reference. Gray arrows and thick black lines above the alignments indicate gene orientations. White peaks represent differences among CP genomes. Exons, introns, and conserved noncoding sequences (CNSs) were displayed as different colors. A similarity cut-off value of 70% was used for the plots, and the Y-axis represents the percentage similarity (50–100%)

The structure of the plant CP genome is highly conserved, highly mutated regions can be easily identified by comparative analyses [16]. These highly variable regions help to elucidate the genetic structure and evolutionary relationships of plants in different environments [59, 60]. In order to evaluate the CP genome differences of the *Lasianthus* species, we downloaded the complete CP genome (.fas) format sequences of six *Lasianthus* species from the NCBI database. Then, we combined the CP genome information of *L. attenuatus*, *L. henryi*, *L. hookeri* and *L. sikkimensis* with six CP genomes (*L. chrysoneurus*, *L. hookeri* var. *dunnianus*, *L. japonicus*, *L. rigidus*, *L. verticillatus*, *L. sp.*) of *Lasianthus* species published in the NCBI database. We performed comparisons and analyses using mVISTA software with *L. attenuatus* as the reference sequence (Fig. 5). These analyses revealed that, except for *L. sp*., the *Lasianthus* species CP genome sequences had little difference. The sequence differences were mainly concentrated in the non-coding region, while the exon and untranslated region (UTR) had only slight differences between the genomes. The most differentiated non-coding regions include *acc*D-*psa*I, *psa*I-*ycf*4, *rbc*L-*acc*D, *ycf*4-*cem*A, *ndh*C-*trn*V-UAC, *pet*A-*psb*J-*psb*L, *trn*E-UUC- *trn*T-GGU and *trn*T-*trn*L. Furthermore, we found that the most of the sequence variation was in the LSC and SSC regions, with the smallest sequence variation in the IR region. This result further supports the idea that the coding regions are more conservative than the non-coding regions, and that the IR regions are more conserved than the LSC and SSC regions in higher plants [14, 61, 62]. This phenomenon may be due to gene conversion correcting mutations in the IR sequence [63].

**Fig. 6:**
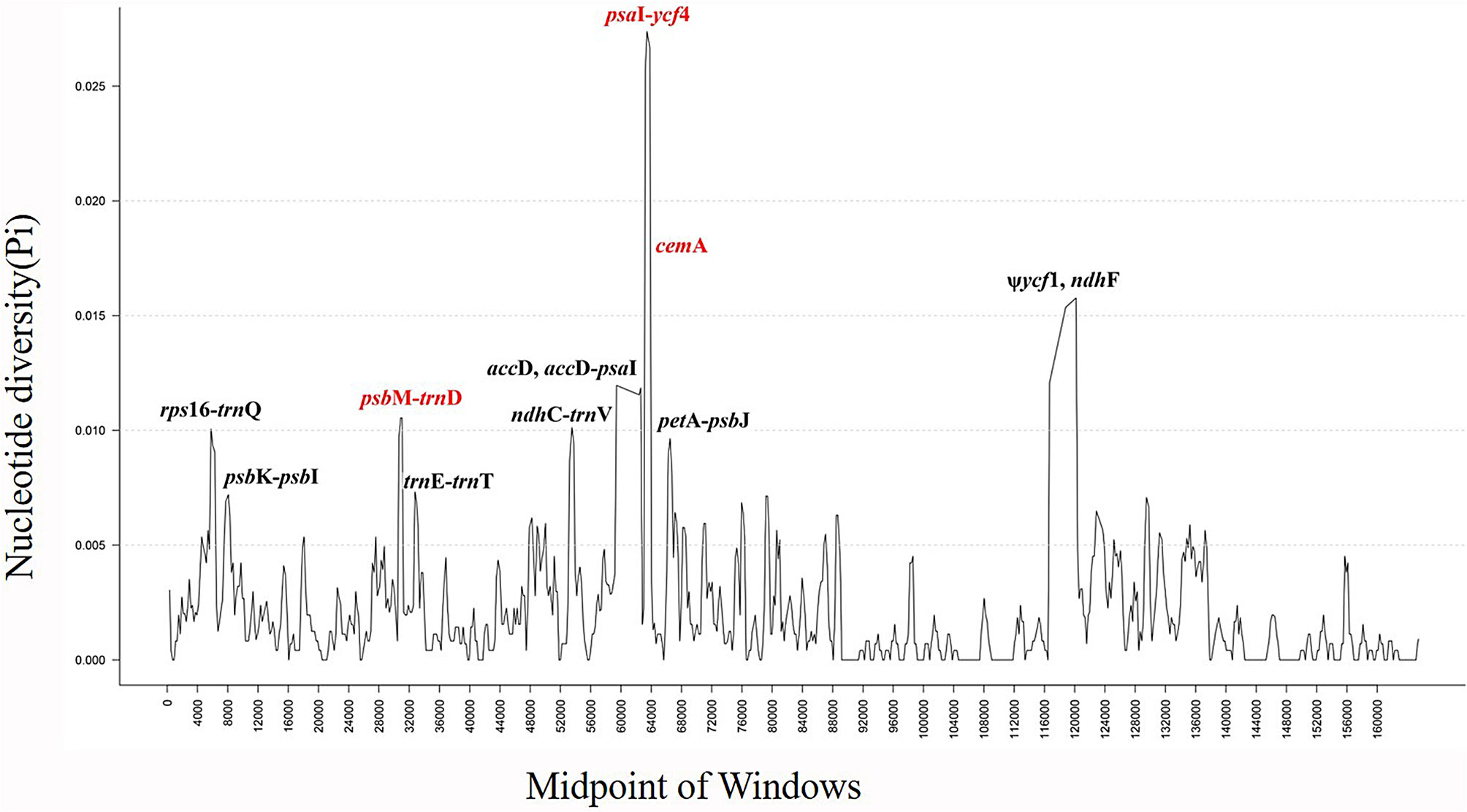
Nucleotide diversity (Pi) values analysis based on the complete chloroplast (CP) genomes of ten *Lasianthus* species. Window length: 600 bp; step size: 200 bp. X-axis: position of the midpoint of a window. Y-axis: nucleotide diversity of each window

Next, the nucleotide diversity (Pi) and the highly variable regions of whole CP genome sequence in *Lasianthus* were detected by using DnaSP [31] software (Fig. 6). The IR region exhibits lower variability than the LSC and SSC regions. The test results showed that the Pi average value was 0.002029 (Table S3). Additionally, the Pi values of two highly variable regions in the LSC and SSC regions with Pi value greater than 0.015, were 0.0266 (*psa*I-*ycf*4-*cem*A) and 0.0156 (*ndh*F), respectively. The relatively high Pi values in the LSC and SSC regions indicate that rapid nucleotide substitution may occur during genome evolution, which plays an important role in species identification and phylogenetic analysis.

### Molecular marker development and polymorphism verification

Previous studies on the molecular identification of *Panax*, *Zanthoxylum* and *Alpinia* species showed that CP genetic markers had high identification efficiency [18, 64, 65]. Compared with the whole CP genome, CP barcode fragments have the advantages of low sequencing costs and easy and fast analysis. Therefore, based on the alignment of complete CP genome sequences, 11 highly variable sites were selected as candidate DNA markers for identifying *Lasianthus* species. A total of 35 samples of seven species of *Lasianthus* (*L. attenuatus*, *L. henryi*, *L. hookeri*, *L. sikkimensis*, *L. fordii* var. *trichocladus*, *L. hookeri* var. *dunnianus* and *L. verticillatus*) were collected to verify the identification efficiency of the candidate barcode fragments. The sample number and location information are listed in Table S1. To develop identification markers for species authentication of *Lasianthus*, specific primers for the conserved regions of eleven highly variable sites (*rps*16-*trn*Q, *psb*K-*psb*I, *psb*M-*trn*D, *trn*E-*trn*T, *ndh*C-*trn*V, *acc*D-*psa*I, *psa*I-*ycf*4, *cem*A, *pet*A-*psb*J, *ycf*1, *ndh*F) were designed. Then, five barcode markers were successfully amplified into fragments of the expected sizes, and their PCR products were sent to the Sangon Laboratory for sequencing. Ultimately, sequences for three markers (*psa*I-*ycf*4, *psb*M-*trn*D, *cem*A) were successfully obtained. At the same time, five conventional DNA barcodes ITS, ITS2, *psb*A-*trn*H, *rbc*L and *mat*K were used to amplify seven *Lasianthus* species to evaluate their identification efficiency. The information of conventional barcodes and selected chloroplast molecular marker are shown in Table S4.

**Fig. 7:**
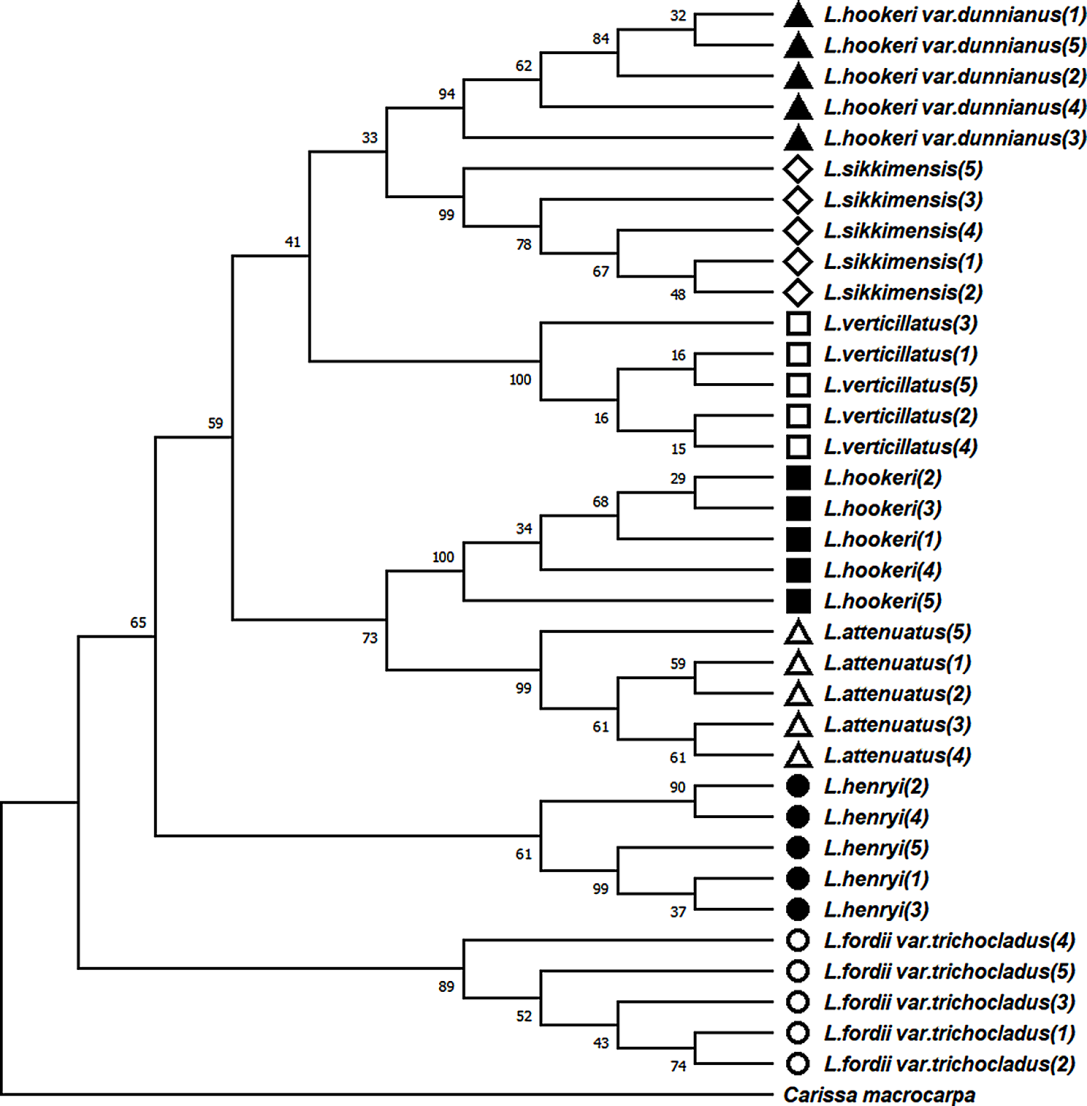
Neighbor-joining bootstrap trees (based on Kimura-2-Parameter) illustrating the resolution of the seven *Lasianthus* species for the “ITS2 + *psa*I-*ycf*4” barcoding

To detect molecular marker polymorphism and determine the most effective *Lasianthus* species identification barcode marker, we analyzed conventional barcodes and selected chloroplast molecular marker parameters, such as average differences length (bp), PCR success rate (%), intraspecific and interspecific differences (%), and average sequence differences between each marker and different markers (refer to Table 3). We constructed an NJ phylogenetic tree using conventional barcodes and screened chloroplast molecular markers. Our analysis revealed that no single fragment among these markers provided sufficient information to distinguish the seven species of *Lasianthus*. Ultimately, we discovered that the ideal combination fragment' ITS2 + *psa*I-*ycf*4 ‘, effectively identifies seven species within the genus (Fig. 7). Additionally, both fragments demonstrated high amplification and sequencing success rates, and the phylogenetic tree constructed using the combined fragments had anticipatively support rates. Based on our results, we suggest that more highly variable regions should be selected as candidate molecular markers, and the combination of two or more markers should be considered for the reliable identification of different species within some specifically genus which could not be authenticated efficiently in future studies. In recent years, numerous studies have utilized chloroplast genomes to detect highly variable regions as molecular markers for species identification, however, this method is still limited to a few taxa and limited samples [18, 66]. Therefore, we suggest that the combination of barcode fragments can be used for species identification for different taxa.

**Table 3:** Characteristics of the different barcode marker loci of seven *Lasianthus* species

	ITS2	*rbc*L	*psb*A-*trn*H	*mat*K	ITS	*cem*A	*psa*I-*ycf*4	*psb*M-*trn*D
Number of successful sequencing/Number of samples	35/35	30/35	33/35	35/35	33/35	35	35	35
Consistent sequence length(bp)	319	515	254	806	851	554	337	535
GC content (%)	59.47%	44.60%	29.26%	33.72%	54.10%	33.47%	22.19%	29.65%
Efficiency of PCR amplification (%)	100%	100%	100%	100%	100%	100%	100%	100%
Success rate of sequencing (%)	100%	85.71%	94%	100%	94%	100%	100%	100%
Parsimony informative sites/No.variable sites	61/69	6/13	5/25	8/8	147/207	4/4	4/4	16/16
No. variable sites/analysis sequence length	69/319 (21.63%)	13/515 (2.52%)	25/254 (9.84%)	8/806 (1%)	207/851 (24.32%)	4/554 (0.7%)	4/337 (1.2%)	16/535 (3%)

### Comparative genomic in Rubiaceae

**Fig. 8:**
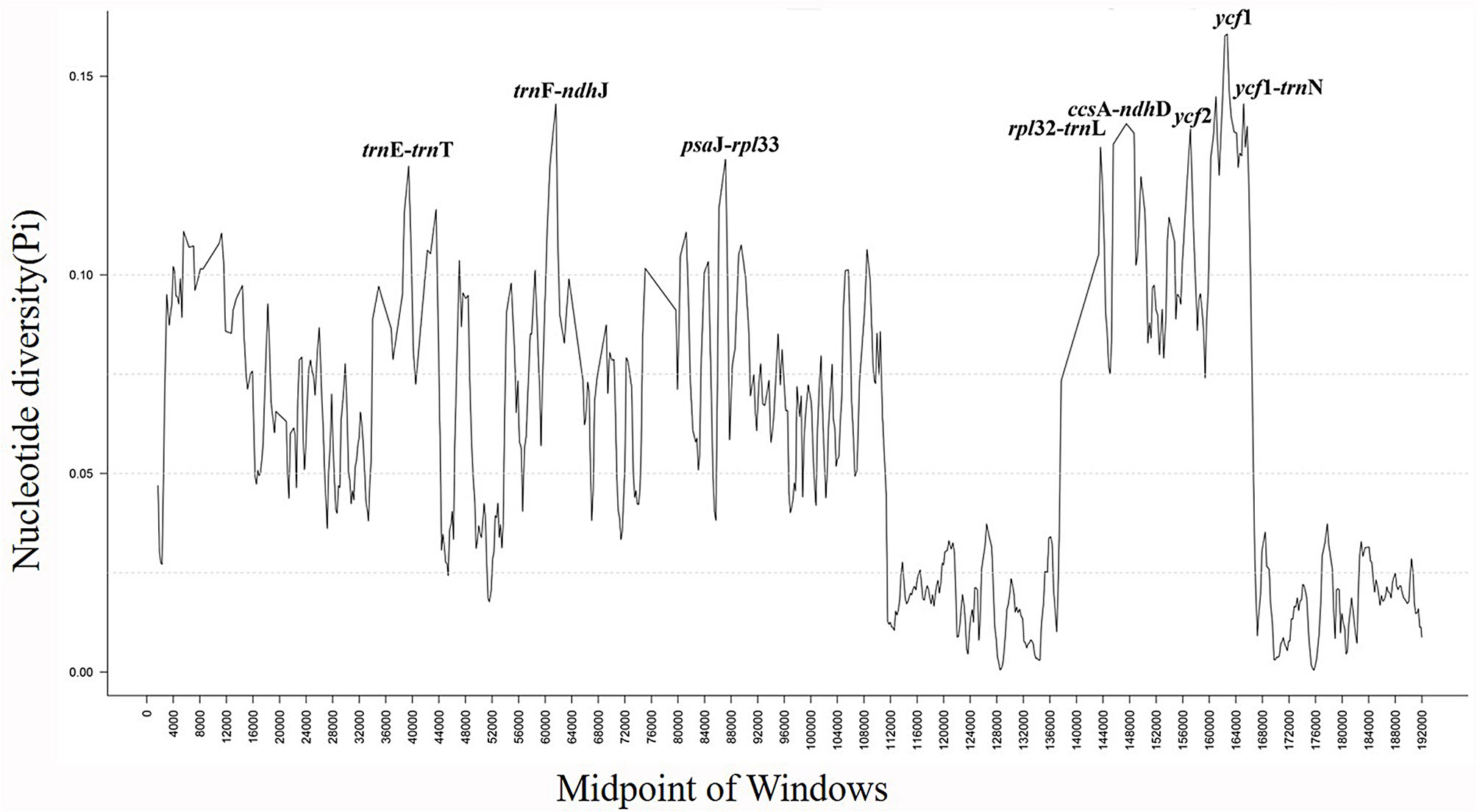
Nucleotide polymorphism (Pi) values analysis based on the complete chloroplast (CP) genomes of 49 Rubiaceae species. Window length: 600 bp; step size: 200 bp. X-axis: position of the midpoint of a window. Y-axis: nucleotide diversity of each window

To detect divergence in the CP genome of the *Lasianthus* species of Rubiaceae, we downloaded 45 species complete CP genome (.fas) format sequences of Rubiaceae from NCBI database (Table S5), and comparative analysis was conducted by aligning the CP genome sequences using *L. attenuatus* as a reference genome (Fig. S1, Fig. 8). The results showed that the chloroplast genome sequences of species in below the genus level are highly similar, but there are great differences among different genera. Thereinto, the most significant variation lies in the *ycf*1 gene, indicating that it was active in the evolution process of chloroplast genomes. Some researchers believe that *ycf*1 is the most variable plastid genome region and can serve as a core barcode of land plants [67]. Kikuchi et al. [68] provided evidence that *ycf*1 is indeed TIC 214, a crucial component of the protein translocon on the inner chloroplast membrane. Meanwhile, an interesting phenomenon was discovered in the comparison of chloroplast genomes Rubiaceae species, with the length of pseudogene *ycf*1 at the IRa / LSC boundary in the chloroplast genome of *Lasianthus* species is significantly longer than that in other genera of Rubiaceae species. Pseudogenes are classically believed to be insignificant and considered as ‘genomic junk’, were reported by Anand et al. [69] to undergo repair of pseudogene *efe*U under a designed selection pressure during adaptive laboratory evolution. This result indicates that some pseudogenes can recover their functions under certain pressures, emphasizing their importance for genome adaptive evolution. In addition, the intergenic region *trn*F-*ndh*J, *ycf*1-*trn*N, *rpl*32-*trn*L, *trn*E-*trn*T and *psa*J-*rpl*33 of *Lasianthus* species were significantly different from other groups in Rubiaceae. Due to the fact that intergenic regions are not directly involved in protein coding, their functional research has rarely reported. In recent years, some researchers have found that intergenic regions drive gene expression, indicating that intergenic regions are closely related to gene transcription regulation [70, 71]. The chloroplast intergenic regions also have great potential in species identification and phylogenetic evolution, which has been verified among species of *Lilium*, *Dracaena* and *Alpinia* [18, 19, 72]. On the whole, the coding region is more conservative than the non-coding region, and IRs are also more conservative than LSC and SSC. We speculate that during genome evolution, the LSC and SSC regions of Rubiaceae species undergo rapid nucleotide substitution. These variation regions are of great significance for species identification and genome adaptive evolution within the Rubiaceae family.

### Phylogenetic relationships in Rubiaceae

**Fig. 9:**
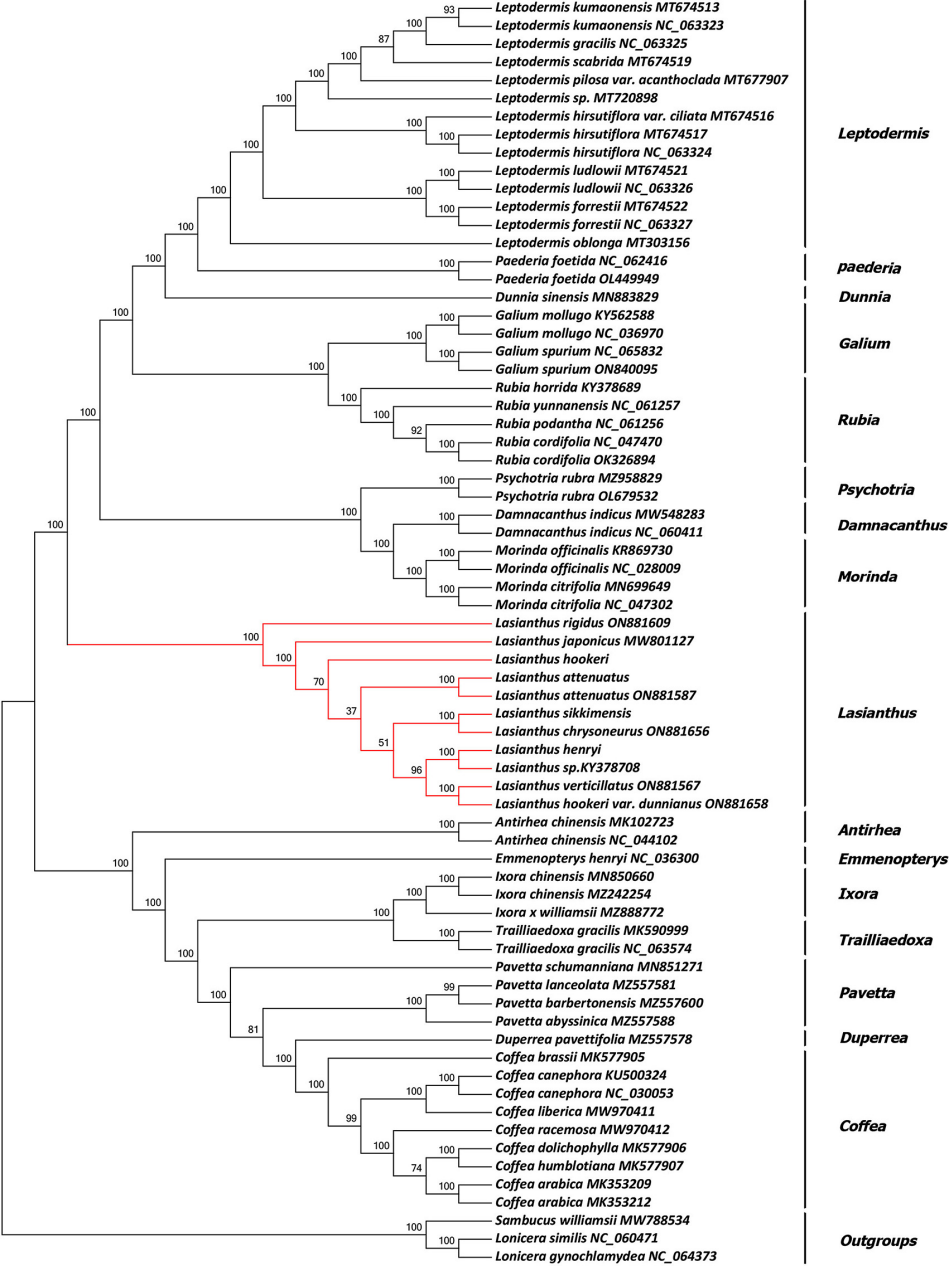
Phylogenetic tree constructed using neighbor-joining based on the 49 species CP genomes of Rubiaceae. Numbers at branch nodes are the bootstrap support values

At present, chloroplast genome sequences are widely used in phylogenetic relationships, genetic structure analysis and species identification of higher plants [73–75]. To explore the phylogenetic relationship of *Lasianthus* and the phylogenetic position of *Lasianthus* in the Rubiaceae family, we obtained 67 complete CP genome sequences belonging to 16 genera of Rubiaceae, and constructed NJ (Fig. 9), ML (Fig. S2), MP (Fig. S3) phylogenetic trees were constructed using *L. gynochlamydea*, *L. similis* and *Sambucus williamsii* as outgroups. Three methods generated nearly identical topology, and all nodes were well supported. The phylogenetic trees showed that 10 species of *Lasianthus* were clustered into one branch, and each species was separated from each other. Thus, we believe that the CP genomes can be used to identify the *Lasianthus* species. The chloroplast genome sequence serves as a super-barcode providing a useful method for species identification of advanced plants. Meanwhile, the 16 genera of Rubiaceae were divided into two large branches: the *Lasianthus*, *Morinda*, *Paederia*, *Psychotria*, *Galium*, *Rubia*, *Dunnia*, *Leptodermis* and *Damnacanthus* were grouped together in one large branch, whereas the remaining species are included in another large branch. Then, the ten species of *Lasianthus* split off into a branch, and the remaining eight genera species split off into another large branch. Rubiaceae includes approximately 700 species of 97 genera in China, and *Lasianthus* includes over 200 species. Therefore, for a clearer understanding of the species relationships in Rubiaceae and *Lasianthus*, future phylogenetic analyses should include more CP genome samples.

## Conclusion

In this study, we reveal the detailed characteristics of the complete CP genome of four *Lasianthus* species, and the gene order, SSRs, GC content and IR/SC boundary structure were highly similar. we then combined these data with publicly available CP genome data from six other *Lasianthus* species to compared the CP genome sequences. Three highly variable regions (*psa*I-*ycf*4, *psb*M-*trn*D, *cem*A) were identified as valuable molecular markers, we ultimately determined that the combination fragment' ITS2 + *psa*I-*ycf*4' is the optimal barcode combination for identifying the genus of *Lasianthus*. Comparative analysis of chloroplast genome of Rubiaceae showed that the coding region is more conservative than the non-coding region, and IRs are also more conservative than LSC and SSC. Finally, the most comprehensive phylogenetic tree to date has been constructed for the Rubiaceae family. These findings provide an important reference point to further studies in the species identification, genetic diversity, and phylogenetic analyses of Rubiaceae species.

## Electronic supplementary material

Below is the link to the electronic supplementary material.

Supplementary Material 1

Supplementary Material 2

Supplementary Material 3

Supplementary Material 4

Supplementary Material 5

Supplementary Material 6

Supplementary Material 7

Supplementary Material 8

## Data Availability

The complete CP genome sequences of *L. attenuatus*, *L. henryi*, *L. hookeri* and *L. sikkimensis* are available in the GenBank with accession numbers of OR490208, OR490209, OR490210 and OR490211, respectively.

## Abbreviations

CP: Chloroplasts
LSC: Large single-copy region
SSC: Small single-copy region
IRs: Inverted repeat regions
SSRs: Simple sequence repeats
RSCU: Relative synonymous codon usage
GC: Guanine-cytosine
Pi: Nucleotide diversity
ML: Maximum Likelihood
NJ: Neighbor-Joining
MP: Maximum Parsimony
CNSs: Conserved noncoding sequences

## References

[1] Arshed MJC, Alejandro GJD. A new Philippine endemic species and new records of *Lasianthus* (Lasiantheae, Rubiaceae). Phytotaxa. 2016;288:296–300. 10.11646/phytotaxa.288.3.12.

[2] Tan MA, Lagamayo MWD, Alejandro GJD, An SSA. Neuroblastoma SHSY5Y cytotoxicity, antiamyloidogenic activity and cyclooxygenase inhibition of *Lasianthus Trichophlebus* (Rubiaceae). 3 Biotech. 2020;10:152. 32181114 10.1007/s13205-020-2145-2 PMC7054575

[3] Yin CY, Yu J, Tang DY, Li HT, Li YH, Li G, Liu SF, Li XL, Mou Y. Investigation on the medicinal and edible plant resources of Dai nationality in Xishuangbanna. Biotic Resour. 2021;43(4):341.

[4] Ong HC, Faezah AW, Milow P. Medicinal plants used by the Jah Hut Orang Asli at Kampung Pos Penderas, Pahang, Malaysia. Ethno Med. 2012;6(1):11–5.

[5] Li B, Zhang DM, Luo YM, Chen XG. Three New and Antitumor Anthraquinone glycosides from *Lasianthus Acuminatissimus* MERR. Chem Pharm Bull. 2006;54(3):297–300. 10.1248/cpb.54.297 16508180

[6] Al-Hamoud GA, Orfali SR, Perveen S, Mizuno K, Takeda Y, Nehira T, Masuda K, Sugimoto S, Yamano Y, Otsuka H, Matsunami K. Lasianosides A–E: New Iridoid glucosides from the leaves of *Lasianthus Verticillatus* (Lour.) Merr. And their antioxidant activity. Molecules. 2019;24:3995. 31694179 10.3390/molecules24213995 PMC6864479

[7] Yu BW, Ge YC, Shi RJ, Ye T, Wu YH, Huo WZ. Chemical constituents from *Lasianthus Wallichii*. J Chin Med Mater. 2019;42(7):1550–3.

[8] Cai M, Zhu H, Wang H. Pollen morphology of the genus *Lasianthus* (Rubiaceae) and related taxa from Asia. J Syst Evol. 2008;46(1):62–72.

[9] Cai M. Study on Micromorphological Characteristics of Lasianthus from Rubiaceae. Xishuangbanna Tropical Botanical Garden. Chinese Academy of Sciences; 2006.

[10] Arshed MJC, Valdez MB, Alejandro GJD. Evaluating the feasibility of five candidate DNA barcoding loci for Philippine *Lasianthus* Jack (*Lasiantheae: Rubiaceae*). Pharmacogn Mag. 2017;13(52):553–8. 29200712 10.4103/pm.pm_1_17 PMC5701390

[11] Sato S, Nakamura Y, Kaneko T, Asamizu E, Tabata S. Complete structure of the Chloroplast Genome of *Arabidopsis thaliana*. DNA Res. 1999;6:283–90. 10574454 10.1093/dnares/6.5.283

[12] Ferrarini M, Moretto M, Ward JA, Surbanovski N, Stevanovic V, Giongo L, Viola R, Cavalieri D, Velasco R, Cestaro A, Sargent DJ. An evaluation of the PacBio RS platform for sequencing and *de novo* assembly of a chloroplast genome. BMC Genom. 2013;14:670. 10.1186/1471-2164-14-670 PMC3853357 24083400

[13] Ahmad W, Asaf S, Khan A, Al-Harrasi A, Al-Okaishi A, Khan AL. Complete chloroplast genome sequencing and comparative analysis of threatened dragon trees *Dracaena serrulata* and *Dracaena cinnabari*. Sci Rep. 2022;12(1):16787. 36202844 10.1038/s41598-022-20304-6 PMC9537188

[14] Fan ZF, Ma CL. Comparative chloroplast genome and phylogenetic analyses of Chinese *Polyspora*. Sci Rep. 2022;12(1):15984. 36163343 10.1038/s41598-022-16290-4 PMC9512918

[15] Wicke S, Schneeweiss GM, dePamphilis CW, Müller KF, Quandt D. The evolution of the plastid chromosome in land plants: gene content, gene order, gene function. Plant Mol Biol. 2011;76:273–97. 21424877 10.1007/s11103-011-9762-4 PMC3104136

[16] Hong Z, Wu ZQ, Zhao KK, Yang ZJ, Zhang NN, Guo JY, Tembrock LR, Xu DP. Comparative analyses of five complete chloroplast genomes from the Genus *Pterocarpus* (Fabacaeae). Int J Mol Sci. 2020;21:3758. 32466556 10.3390/ijms21113758 PMC7312355

[17] Chen XL, Zhou JG, Cui YX, Wang Y, Duan BZ, Yao H. Identification of *Ligularia* herbs using the complete chloroplast genome as a Super-barcode. Front Pharmacol. 2018;9:695. 30034337 10.3389/fphar.2018.00695 PMC6043804

[18] Yang HY, Wang LQ, Chen HM, Jiang M, Wu WW, Liu SY, Wang JH, Liu C. Phylogenetic analysis and development of molecular markers for five medicinal *Alpinia* species based on complete plastome sequences. BMC Plant Biol. 2021;21:431. 34551721 10.1186/s12870-021-03204-1 PMC8456601

[19] Zhang Y, Song MF, Li HT, Sun HF, Zhang ZL. DNA barcoding identification of original plants of a rare medicinal material Resina Draconis and related *Dracaena* species. China J Chin Mater Med. 2021;46:2173–81. 10.19540/j.cnki.cjcmm.20210124.104 34047118

[20] Brown J, Pirrung M, Lee AM. FQC Dashboard: integrates FastQC results into a web-based, interactive, and extensible FASTQ quality control tool. Bioinformatics. 2017;33(19):3137–9. 28605449 10.1093/bioinformatics/btx373 PMC5870778

[21] Jin JJ, Yu WB, Yang JB, Song Y, dePamphilis CW, Yi TS, Li DZ. GetOrganelle: a fast and versatile toolkit for accurate de novo assembly of organelle genomes. Genome Biol. 2020;21:241. 32912315 10.1186/s13059-020-02154-5 PMC7488116

[22] Wick RR, Schultz MB, Zobel J, Holt KE. Bandage: interactive visualization of de novo genome assemblies[J]. Bioinformatics. 2015;31(20):3350–2. 26099265 10.1093/bioinformatics/btv383 PMC4595904

[23] Kearse M, Moir R, Wilson A, Stones-Havas S, Cheung M, Sturrock S, Buxton S, Cooper A, Markowitz S, Duran C, Thierer T, Ashton B, Meintjes P, Drummond A. Geneious Basic: an integrated and extendable desktop software platform for the organization and analysis of sequence data. Bioinformatics. 2012;28(12):1647–9. 22543367 10.1093/bioinformatics/bts199 PMC3371832

[24] Lowe TM, Chan PP. tRNAscan-SE On-line: integrating search and context for analysis of transfer RNA genes. Nucleic Acids Res. 2016;44:W54–7. 27174935 10.1093/nar/gkw413 PMC4987944

[25] Lohse M, Drechsel O, Kahlau S, Bock R. OrganellarGenomeDRAW-a suite of tools for generating physical maps of plastid and mitochondrial genomes and visualizing expression data sets. Nucleic Acids Res. 2013;41:W575–81. 23609545 10.1093/nar/gkt289 PMC3692101

[26] Sharp PM, Li WH. The codon Adaptation Index-a measure of directional synonymous codon usage bias, and its potential applications. Nucleic Acids Res. 1987;15:1281–95. 3547335 10.1093/nar/15.3.1281 PMC340524

[27] Kumar S, Stecher G, Li M, Knyaz C, Tamura K. MEGA X: Molecular Evolutionary Genetics Analysis across Computing platforms. Mol Biol Evol. 2018;35(6):1547–9. 29722887 10.1093/molbev/msy096 PMC5967553

[28] Kurtz S, Choudhuri JV, Ohlebusch E, Schleiermacher C, Stoye J, Giegerich R. REPuter: the manifold applications of repeat analysis on a genomic scale. Nucleic Acids Res. 2001;29:4633–42. 11713313 10.1093/nar/29.22.4633 PMC92531

[29] Katoh K, Rozewicki J, Yamada KD. MAFFT online service: multiple sequence alignment, interactive sequence choice and visualization. Brief Bioinform. 2019;20(4):1160–6. 28968734 10.1093/bib/bbx108 PMC6781576

[30] Frazer KA, Pachter L, Poliakov A, Rubin EM, Dubchak I. VISTA: computational tools for comparative genomics. Nucleic Acids Res. 2004;32:W273–9. 15215394 10.1093/nar/gkh458 PMC441596

[31] Rozas J, Ferrer-Mata A, Sánchez-Delbarrio JC, Guirao-Rico S, Librado P, Ramos-Onsins SE, Sánchez-Gracia A. Mol Biol Evol. 2017;34(12):3299–302. DnaSP 6: DNA Sequence Polymorphism Analysis of Large Data Sets. 10.1093/molbev/msx248 29029172

[32] Darriba D, Posada D, Kozlov AM, Stamatakis A, Morel B, Flouri T. ModelTest-NG: a New and Scalable Tool for the selection of DNA and protein evolutionary models. Mol Biol Evol. 2019;37(1):291–4. 10.1093/molbev/msz189 PMC6984357 31432070

[33] Gu L, Su T, Luo GL, Hu GX. The complete chloroplast genome sequence of *Heteropolygonatum Ginfushanicum* (Asparagaceae) and phylogenetic analysis. Mitochondrial DNA Part B. 2021;6:1799–802. 34104777 10.1080/23802359.2021.1933636 PMC8168753

[34] Chen ZY, Yu XL, Yang YJ, Wei P, Zhang WC, Li XZ, Liu CL, Zhao SQ, Li XY, Liu X. Comparative analysis of Chloroplast genomes within *Saxifraga* (Saxifragaceae) takes insights into their genomic evolution and adaption to the high-elevation environment. Genes (Basel). 2022;13(9):1673. 36140840 10.3390/genes13091673 PMC9498722

[35] Xu C, Cai X, Chen Q, Zhou H, Cai Y, Ben A. Factors affecting synonymous codon usage bias in chloroplast genome of oncidium gower ramsey. Evol Bioinform. 2011;7:271–8. 10.4137/EBO.S8092 PMC3255522 22253533

[36] Das S, Paul S, Dutta C. Synonymous codon usage in adenoviruses: influence of mutation, selection and protein hydropathy. Virus Res. 2006;117(2):227–36. 16307819 10.1016/j.virusres.2005.10.007

[37] Zhang Y, Song MF, Li Y, Sun HF, Tang DY, Xu AS, Yin CY, Zhang ZL, Zhang LX. Complete Chloroplast Genome Analysis of Two Important Medicinal Alpinia Species: *Alpinia galanga* and *Alpinia kwangsiensis*. Front Plant Sci. 2021;12:705892. 34975932 10.3389/fpls.2021.705892 PMC8714959

[38] Kim KJ, Lee HL. Complete chloroplast genome sequences from Korean ginseng (*Panax schinseng* Nees) and comparative analysis of sequence evolution among 17 vascular plants. DNA Res. 2004;11(4):247–61. 15500250 10.1093/dnares/11.4.247

[39] Zhang P, Xu W, Lu X, Wang L. Analysis of codon usage bias of chloroplast genomes in Gynostemma species. Physiol Mol Biol Plants. 2021;27(12):2727–37. 35035132 10.1007/s12298-021-01105-z PMC8720125

[40] Huang S, Kang ZJ, Chen ZF, Deng YF. Comparative analysis of the Chloroplast Genome of *Cardamine hupingshanensis* and phylogenetic study of *Cardamine*. Genes (Basel). 2022;13(11):2116. 36421792 10.3390/genes13112116 PMC9690686

[41] Li Y, Kuang XJ, Zhu XX, Zhu YJ, Sun C. Codon usage bias of Catharanthus roseus. China J Chin Mater Med. 2016;41(22):4165–8. 10.4268/cjcmm20162213 28933083

[42] Gao BM, Yuan L, Tang TL, Hou J, Pan K, Wei N. The complete chloroplast genome sequence of *Alpinia Oxyphylla* Miq. And comparison analysis within the Zingiberaceae family. PLoS ONE. 2019;14(6):e0218817. 31233551 10.1371/journal.pone.0218817 PMC6590956

[43] Ivanova Z, Sablok G, Daskalova E, Zahmanova G, Apostolova E, Yahubyan G, Baev V. Chloroplast Genome Analysis of Resurrection Tertiary Relict *Haberlea rhodopensis* highlights genes important for desiccation stress response. Front Plant Sci. 2017;8:204. 28265281 10.3389/fpls.2017.00204 PMC5316520

[44] Zuo LH, Shang AQ, Zhang S, Yu XY, Ren YC, Yang MS, Wang JM. The first complete chloroplast genome sequences of *Ulmus* species by *de novo* sequencing: genome comparative and taxonomic position analysis. PLoS ONE. 2017;12(2):e0171264. 28158318 10.1371/journal.pone.0171264 PMC5291543

[45] Marechal A, Brisson N. Recombination and the maintenance of plant organelle genome stability. New Phytol. 2010;186(2):299–317. 20180912 10.1111/j.1469-8137.2010.03195.x

[46] Ebert D, Peakall R. Chloroplast simple sequence repeats (cpSSRs): technical resources and recommendations for expanding cpSSR discovery and applications to a wide array of plant species. Mol Ecol Res. 2009;9(3):673–90. 10.1111/j.1755-0998.2008.02319.x 21564725

[47] Kuang DY, Wu H, Wang YL, Gao LM, Zhang SZ, Lu L. Complete chloroplast genome sequence of *Magnolia kwangsiensis* (Magnoliaceae): implication for DNA barcoding and population genetics. Genome. 2011;54:663–73. 21793699 10.1139/g11-026

[48] Wang YF, Wen F, Hong X, Li ZL, Mi YL, Zhao B. Comparative chloroplast genome analyses of *Paraboea* (Gesneriaceae): insights into adaptive evolution and phylogenetic analysis. Front Plant Sci. 2022;13:1019831. 36275537 10.3389/fpls.2022.1019831 PMC9581172

[49] Flannery ML, Mitchell FJ, Coyne S, Kavanagh TA, Burke JI, Salamin N. Plastid genome characterisation in Brassica and Brassicaceae using a new set of nine SSRs. Theor Appl Genet. 2006;113:1221–31. 16909279 10.1007/s00122-006-0377-0

[50] Alzahrani DA, Albokhari EJ, Yaradua SS, Abba A. Comparative analysis of chloroplast genomes of four medicinal capparaceae species: genome structures, phylogenetic relationships and adaptive evolution. Plants. 2021;10:1229. 34204211 10.3390/plants10061229 PMC8234754

[51] Zhou JG, Cui YX, Chen XL, Li Y, Xu ZC, Duan BZ, Li YH, Song JY, Yao H. Complete chloroplast genomes of Papaver rhoeas and Papaver orientale: molecular structures, comparative analysis and phylogenetic analysis. Molecules. 2018;23:437. 29462921 10.3390/molecules23020437 PMC6017017

[52] Asaf S, Waqas M, Khan AL, Khan MA, Kang SM, Imran QM, Shahzad R, Bilal S, Yun BW, Lee IJ. The complete chloroplast genome of Wild Rice (*Oryza minuta*) and its comparison to related species. Front Plant Sci. 2017;8:304. 28326093 10.3389/fpls.2017.00304 PMC5339285

[53] Song WC, Chen ZM, He L, Feng Q, Zhang HR, Du GL, Shi C, Wang S. Comparative Chloroplast Genome Analysis of Wax Gourd (*Benincasa hispida*) with three Benincaseae species, revealing Evolutionary dynamic patterns and phylogenetic implications. Genes (Basel). 2022;13(3):461. 35328015 10.3390/genes13030461 PMC8954987

[54] Zhang YJ, Du LW, Liu A, Chen JJ, Wu L, Hu WM, Zhang W, Kim K, Lee SD, Yang TJ, Wang Y. The Complete Chloroplast Genome Sequences of Five *Epimedium* Species: lights into phylogenetic and taxonomic analyses. Front Plant Sci. 2016;7:306. 27014326 10.3389/fpls.2016.00306 PMC4791396

[55] Lu QX, Chang X, Gao J, Wu X, Wu J, Qi ZC, Wang RH, Yan XL, Li P. Evolutionary comparison of the complete chloroplast genomes in *Convallaria* Species and phylogenetic study of Asparagaceae. Genes (Basel). 2022;13(10):1724. 36292609 10.3390/genes13101724 PMC9601677

[56] Szczecinska M, Sawicki J. Genomic resources of three *Pulsatilla* species reveal evolutionary hotspots, species-specific sites and Variable Plastid structure in the Family Ranunculaceae. Int J Mol Sci. 2015;16:22258–79. 26389887 10.3390/ijms160922258 PMC4613307

[57] Wang W, Messing J. High-throughput sequencing of three *lemnoideae* (duckweeds) chloroplast genomes from. PLoS ONE. 2011;6(9):e24670. 21931804 10.1371/journal.pone.0024670 PMC3170387

[58] Pei JL, Wang Y, Zhuo J, Gao HB, Vasupalli N, Hou D, Lin XC. Complete chloroplast genome features of Dendrocalamusfarinosus and its comparison and evolutionary analysis with other Bambusoideae Species. Genes (Basel). 2022;13(9):1519. 36140690 10.3390/genes13091519 PMC9498922

[59] Daniell H, Lin CS, Yu M, Chang WJ. Chloroplast genomes: diversity, evolution, and applications in genetic engineering. Genome Biol. 2016;17:134. 27339192 10.1186/s13059-016-1004-2 PMC4918201

[60] Abdullah MF, Shahzadi I, Waseem S, Mirza B, Ahmed I, Waheed MT. Chloroplast genome of *Hibiscus rosa-sinensis* (Malvaceae): Comparative analyses and identification of mutational hotspots. Genomics. 2020; 112(1): 581–591. 10.1016/j.ygeno.2019.04.010 30998967

[61] Nazareno AG, Carlsen M, Lohmann LG. Complete chloroplast genome of *Tanaecium Tetragonolobum*: the first Bignoniaceae plastome. PLoS ONE. 2015;10(6):e0129930. 26103589 10.1371/journal.pone.0129930 PMC4478014

[62] Cui YX, Chen XL, Nie LP, Sun W, Hu HY, Lin YL, Li HT, Zheng XL, Song JY, Yao H. Comparison and Phylogenetic Analysis of Chloroplast Genomes of Three Medicinal and Edible *Amomum* Species. Int J Mol Sci. 2019;20(16):4040. 31430862 10.3390/ijms20164040 PMC6720276

[63] Khakhlova O, Bock R. Elimination of deleterious mutations in plastid genomes by gene conversion. Plant J. 2006;46:85–94. 16553897 10.1111/j.1365-313X.2006.02673.x

[64] Lee HJ, Koo HJ, Lee JH, Lee SC, Lee DY, Giang VNL, Kim M, Shim H, Park JY, Yoo KO, Sung SH, Yang TJ. Authentication of *Zanthoxylum* Species Based on Integrated Analysis of Complete Chloroplast Genome Sequences and metabolite profiles. J Agric Food Chem. 2017;65(47):10350–9. 29058421 10.1021/acs.jafc.7b04167

[65] Nguyen VB, Park HS, Lee SC, Lee J, Park JY, Yang TJ. Authentication markers for five major Panax species developed via comparative analysis of complete chloroplast genome sequences. J Agric Food Chem. 2017;65(30):6298–306. 28530408 10.1021/acs.jafc.7b00925

[66] Zhou Y, Nie J, Xiao L, Hu Z, Wang B. Comparative Chloroplast Genome Analysis of Rhubarb Botanical Origins and the development of specific identification markers. Molecules. 2018;23(11):2811. 30380708 10.3390/molecules23112811 PMC6278470

[67] Dong WP, Xu C, Li CH, Sun JH, Zuo YJ, Shi S, Cheng T, Guo JJ, Zhou SL. ycf1, the most promising plastid DNA barcode of land plants. Sci Rep. 2015;12(5):8348. 10.1038/srep08348 PMC4325322 25672218

[68] Kikuchi S, Bédard J, Hirano M, Hirabayashi Y, Oishi M, Imai M, Takase M, Ide T, Nakai M. Uncovering the Protein Translocon at the Chloroplast Inner Envelope membrane. Science. 2013;339:571–4. 23372012 10.1126/science.1229262

[69] Anand A, Olson CA, Yang L, Sastry AV, Catoiu E, Choudhary KS, Phaneuf PV, Sandberg TE, Xu S, Hefner Y, Szubin R, Feist AM, Palsson BO. Pseudogene repair driven by selection pressure applied in experimental evolution. Nat Microbiol. 2019;4(3):386–9. 30692668 10.1038/s41564-018-0340-2

[70] Bondino HG, Valle EM. A small intergenic region drives exclusive tissue-specific expression of the adjacent genes in Arabidopsis thaliana. BMC Mol Biol. 2009;10:95. 19835620 10.1186/1471-2199-10-95 PMC2772851

[71] Mitra A, Han JG, Zhang ZJ, Mitra A. The intergenic region of *Arabidopsis thaliana cab*1 and *cab*2 divergent genes functions as a bidirectional promoter. Planta. 2009;229(5):1015–22. 19169705 10.1007/s00425-008-0859-1

[72] Liu YX, Zhang MF, Chen XQ, Chen X, Hu Y, Gao JL, Pan WQ, Xin Y, Wu J, Du YP, Zhang XH. Developing an efficient DNA barcoding system to differentiate between *Lilium* species. BMC Plant Biol. 2021;21(1):465. 34645404 10.1186/s12870-021-03229-6 PMC8513328

[73] Li HT, Yi TS, Gao LM, Ma PF, Zhang T, Yang JB, Gitzendanner MA, Fritsch PW, Cai J, Luo Y, Wang H, van der Bank M, Zhang SD, Wang QF, Wang J, Zhang ZR, Fu CN, Yang J, Hollingsworth PM, Chase MW, Soltis DE, Soltis PS, Li DZ. Origin of angiosperms and the puzzle of the jurassic gap. Nat Plants. 2019;5(5):461–70. 31061536 10.1038/s41477-019-0421-0

[74] Kyalo CM, Li ZZ, Mkala EM, Malombe I, Hu GW, Wang QF. The first glimpse of *Streptocarpus Ionanthus* (Gesneriaceae) Phylogenomics: analysis of five subspecies’ chloroplast genomes. Plants. 2020;9(4):456. 32260377 10.3390/plants9040456 PMC7238178

[75] Tian XL, Wariss HM. The complete chloroplast genome sequence of *Metabriggsia ovalifolia* W. T. Wang (Gesneriaceae), a national key protected plant endemic to karst areas in China. Mitochondrial DNA B Resour. 2021;6(3):833–4. 33763595 10.1080/23802359.2021.1884021 PMC7954431

