# Supplementary material for: Comprehensive comparative analysis and development of molecular markers for Lasianthus species based on complete chloroplast genome sequences
Source: BMC Plant Biol. 2024 Dec 31;24:867. doi: 10.1186/s12870-024-05383-z (PMC11406864; doi:10.1186/s12870-024-05383-z)
Supplement: Supplementary file 1 — Supplementary Material 1 [file 12870_2024_5383_MOESM1_ESM.docx]

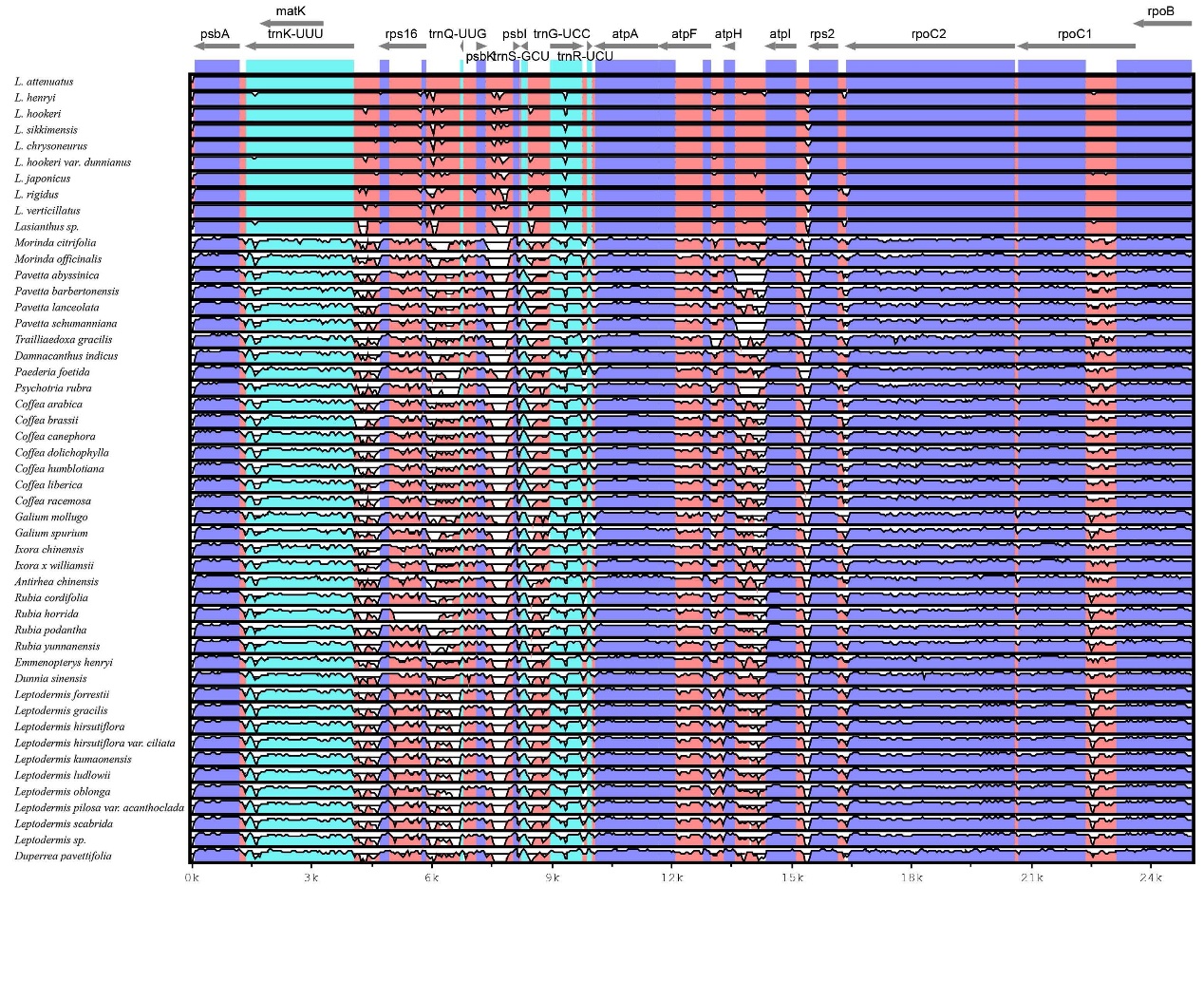

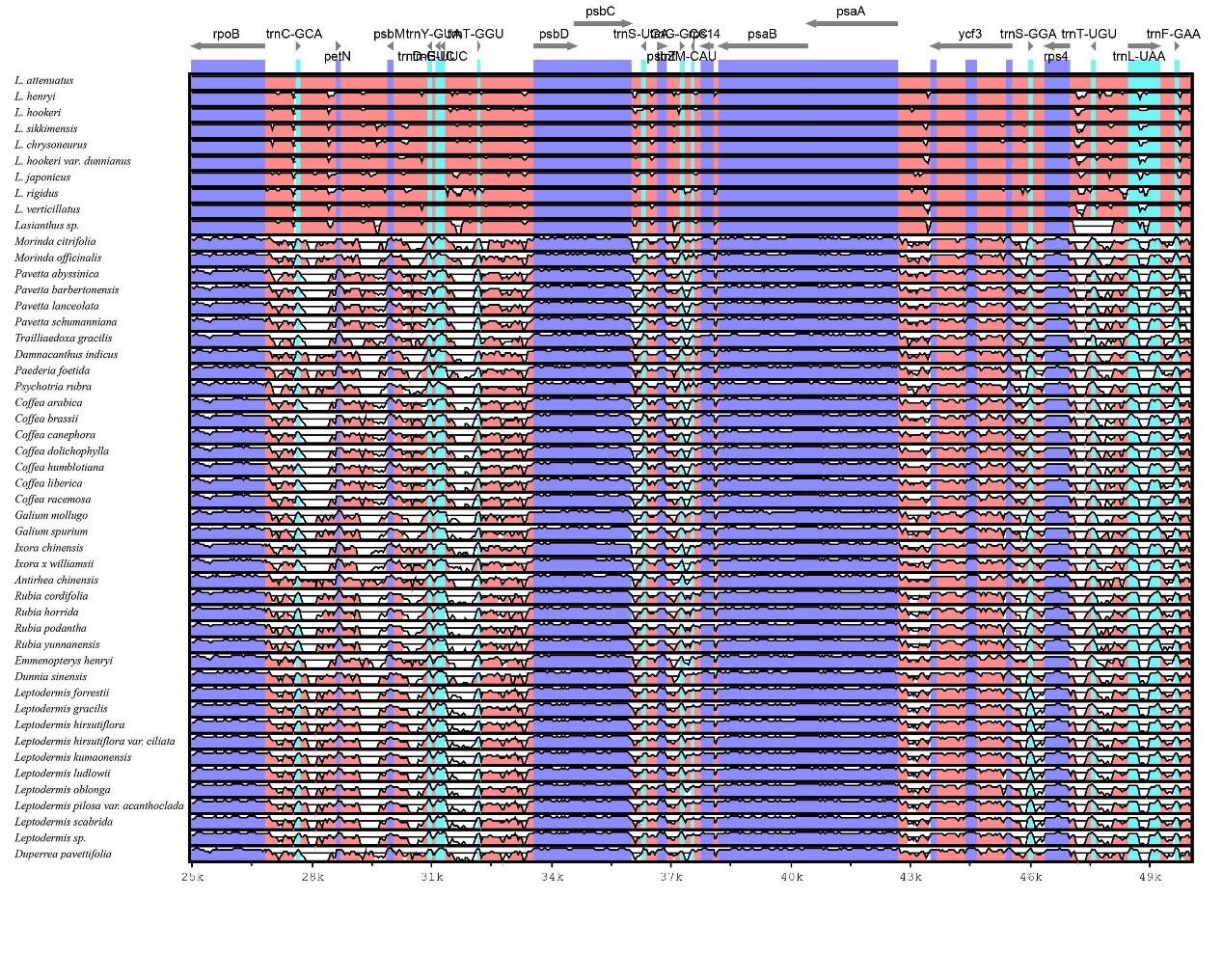

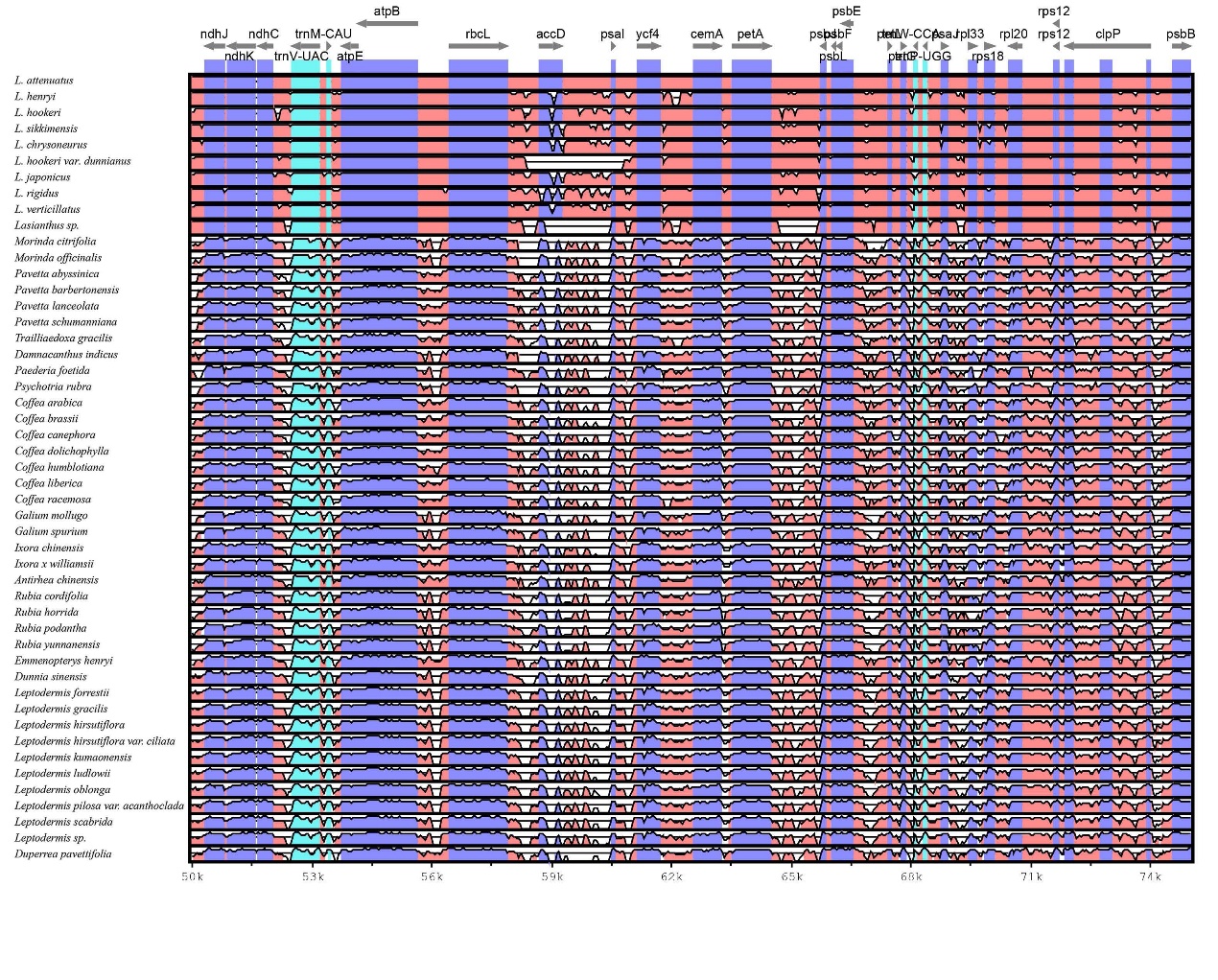

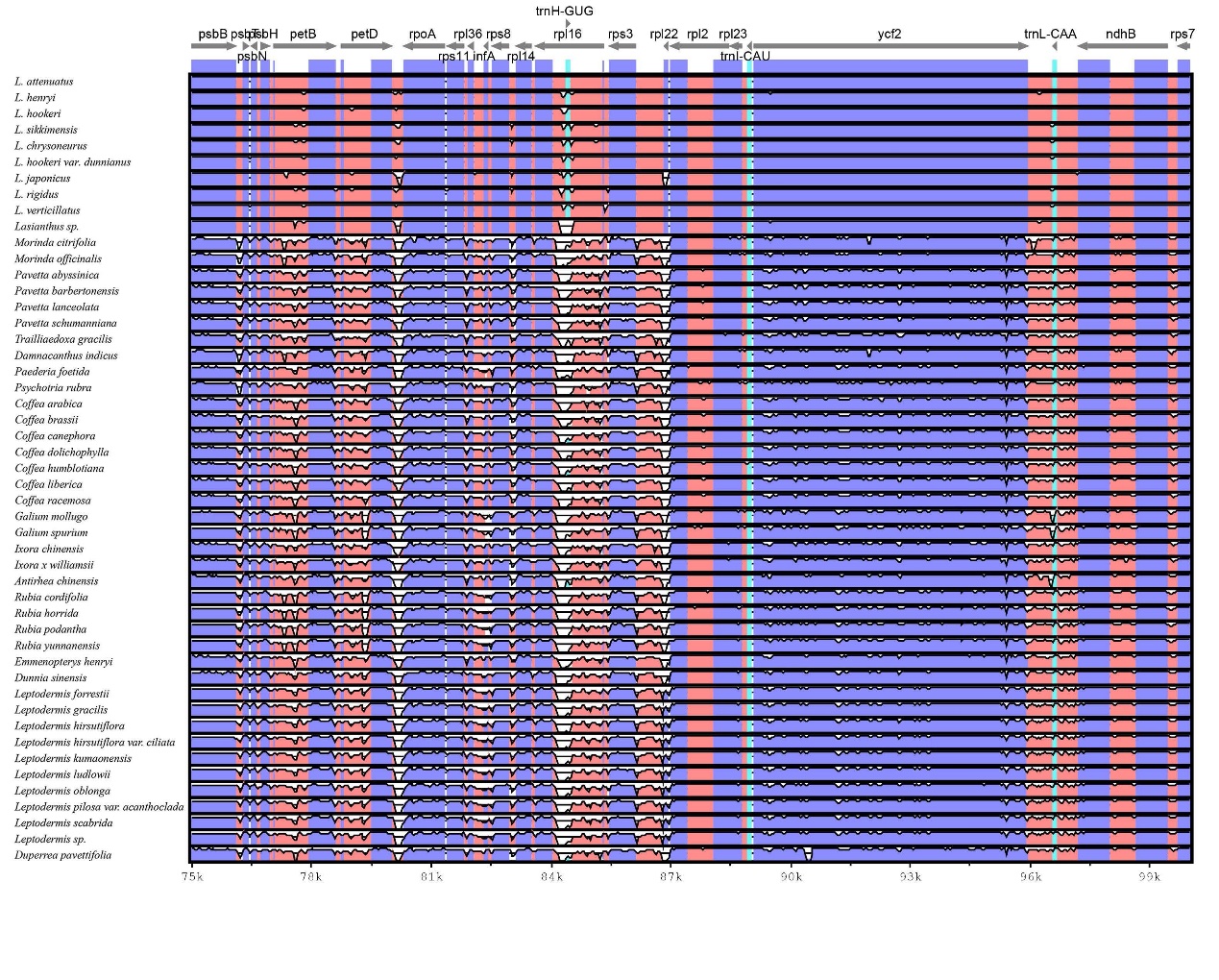

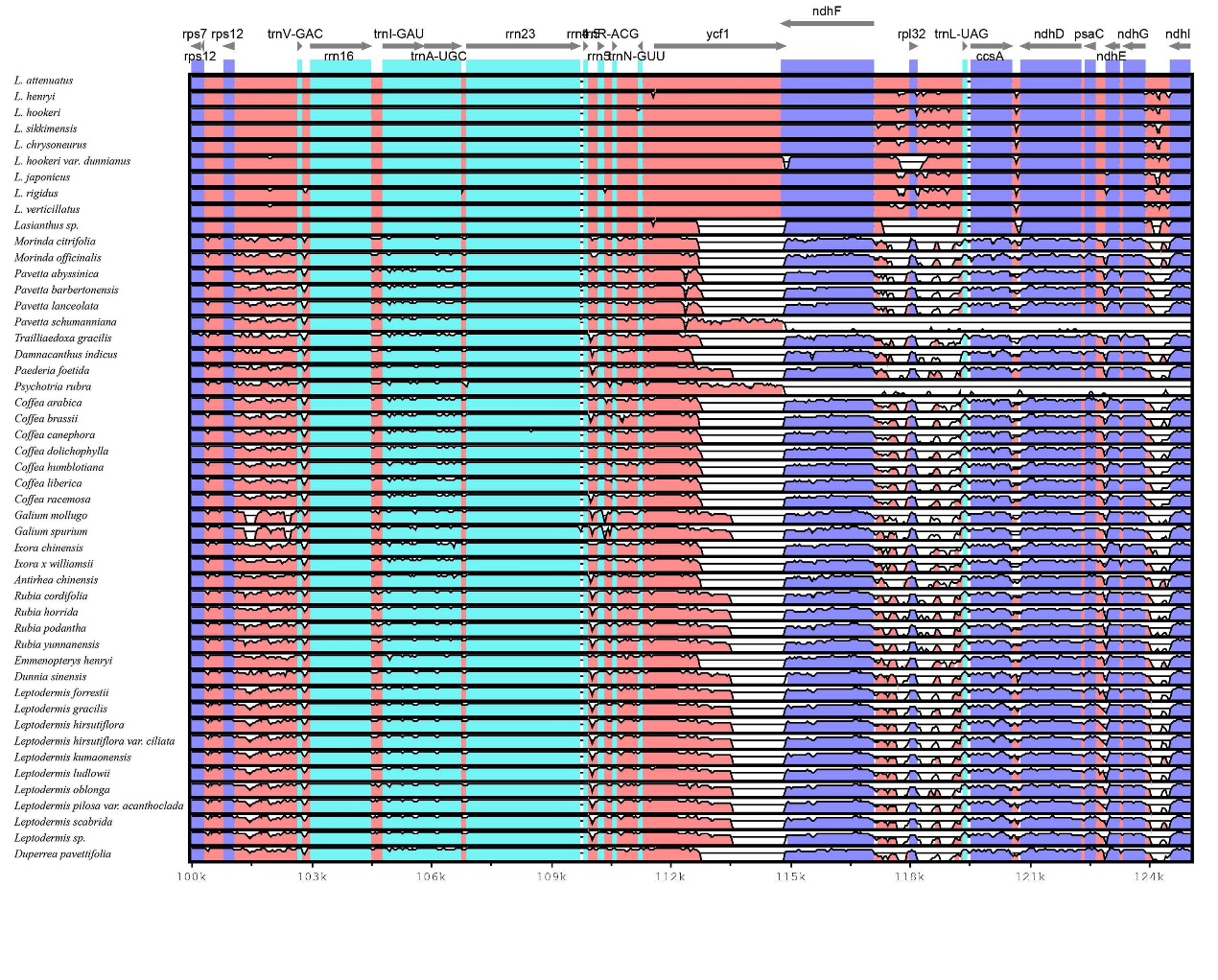

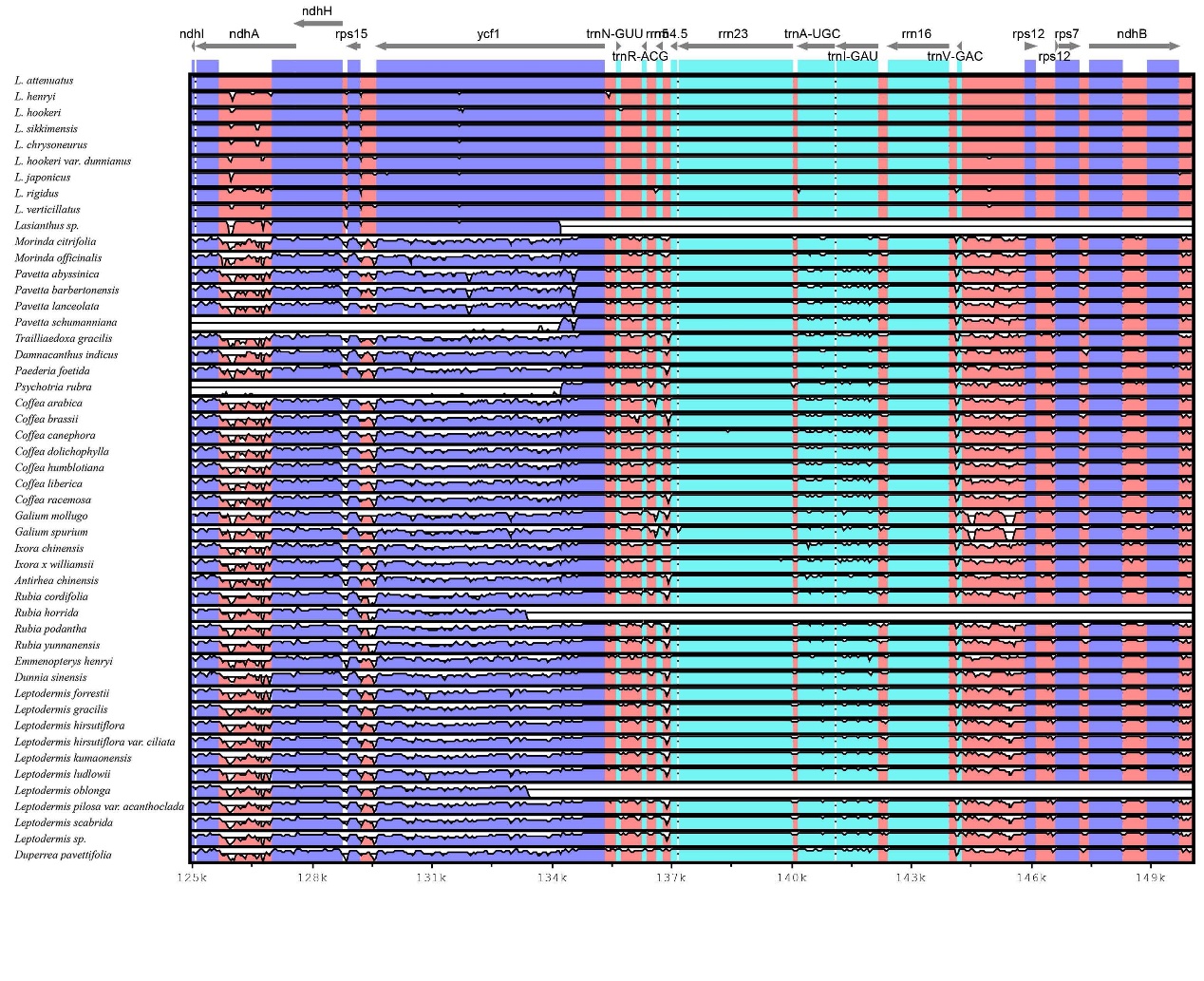

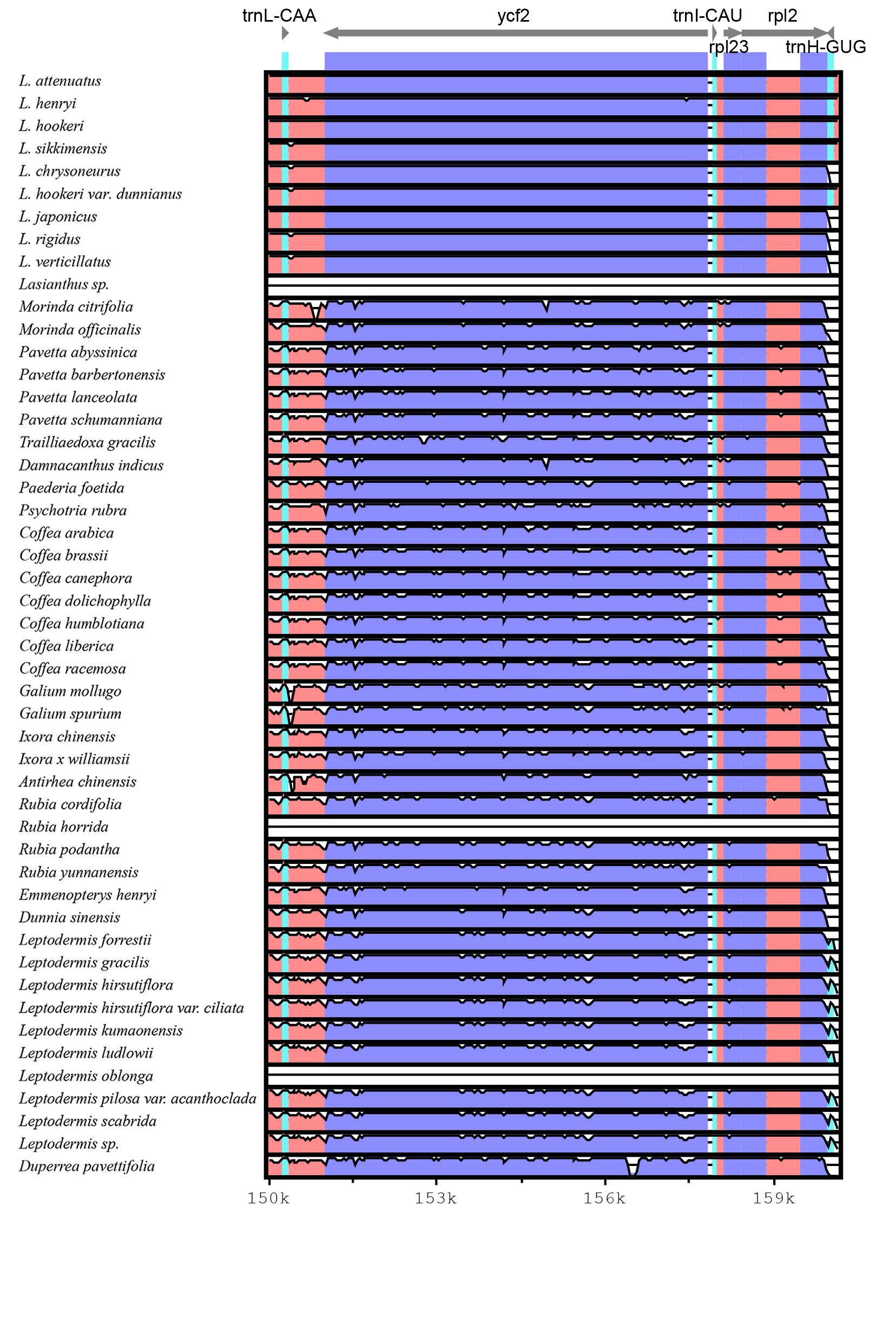


**Fig S1.** The alignment and comparative analysis of the whole CP genome for 49 Rubiaceae species using mVISTA, and using *L. attenuatus* as a reference.
