# Supplementary material for: Comprehensive comparative analysis and development of molecular markers for Lasianthus species based on complete chloroplast genome sequences
Source: BMC Plant Biol. 2024 Dec 31;24:867. doi: 10.1186/s12870-024-05383-z (PMC11406864; doi:10.1186/s12870-024-05383-z)
Supplement: Supplementary file 2 — Supplementary Material 2 [file 12870_2024_5383_MOESM2_ESM.docx]

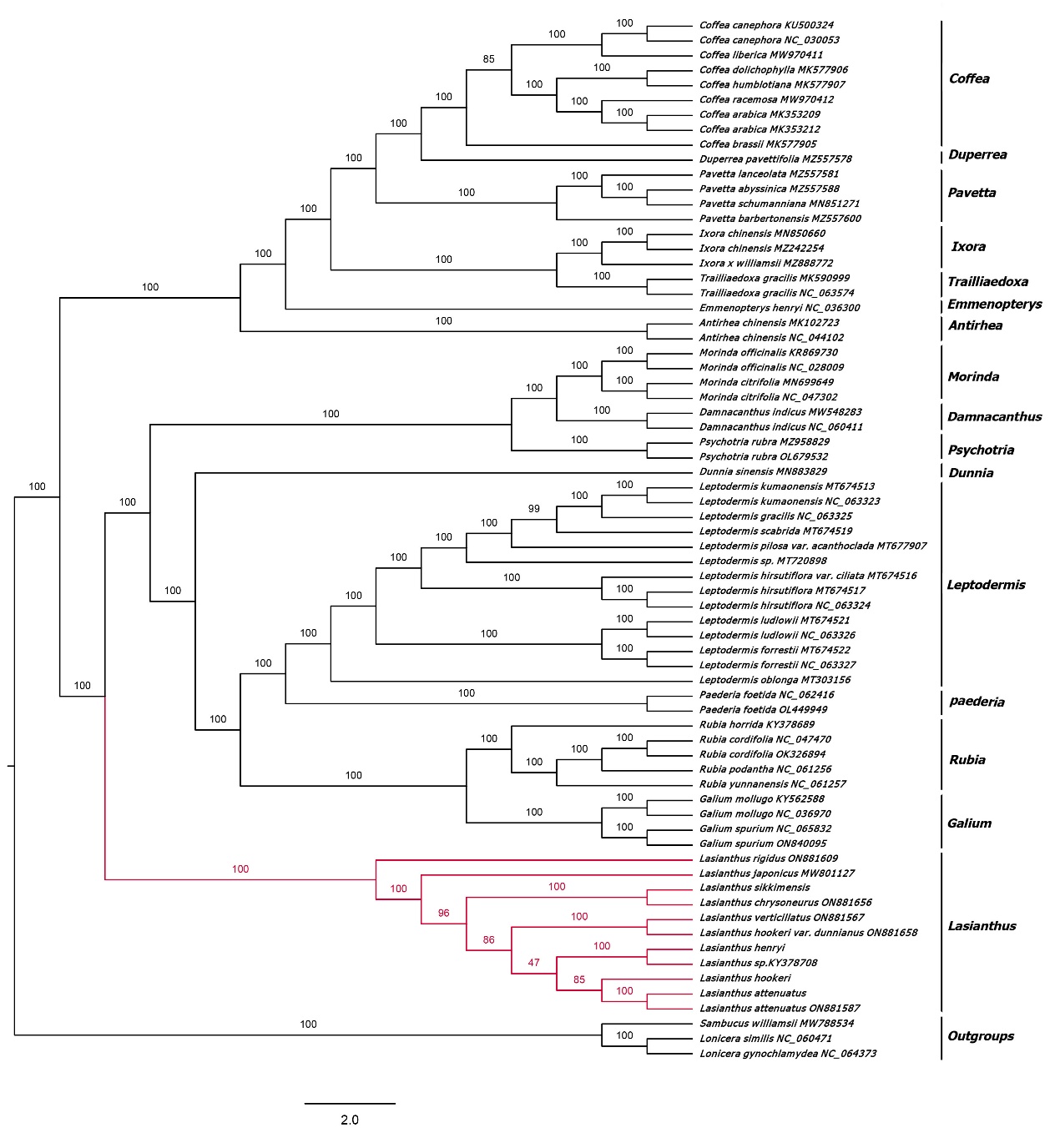


**Fig S2.** Phylogenetic tree constructed using Maximum Likelihood (ML) based on the 49 species CP genomes of Rubiaceae.
